# Putting Emotional Memories in Context: The Constructionist Model of Emotional Memory

**DOI:** 10.1177/17456916261425152

**Published:** 2026-03-10

**Authors:** John T. West, Neil W. Mulligan, Kristen A. Lindquist

**Affiliations:** 1 Department of Psychology, The Pennsylvania State University; 2 Department of Psychology and Neuroscience, University of North Carolina; 3 Department of Psychology, The Ohio State University

**Keywords:** memory, emotion, affect, psychological constructionism

## Abstract

Cognitive psychologists have long been interested in the intersection of emotion and memory, given that the emotions associated with a stimulus affect its memorability. Theoretical perspectives within cognitive science have guided research on how affective dimensions, such as valence and arousal, affect aspects of memory, such as accuracy, subjective vividness, consolidation, and retrieval. Here we argue that well-established theories of emotion from affective science represent a fruitful source of ideas whose implications for episodic memory have not yet been thoroughly investigated. In the current article, we propose a model of emotional memory, inspired by psychological-constructionist theories of emotion, that builds upon existing perspectives in this area while generating several novel hypotheses and avenues of investigation. Following psychological constructionism, we conceive of emotions as emergent phenomena constructed when perceivers use conceptual knowledge to make sense of affective sensations in context. The *constructionist model of emotional memory* (CMEM) highlights new directions for future emotional-memory research, such as investigating the mnemonic consequences of conceptual emotion knowledge and considering the effects of variability in emotion construction at the situational, individual, and cultural levels.

The way that individuals remember emotional events has important consequences for criminal justice, psychopathology, social relationships, and well-being. Consequently, understanding the ways that emotion impacts memory is of great practical as well as theoretical importance. As discussed below, researchers in cognitive science have already devoted decades of work to this topic. This foundational work has contributed significantly to our understanding of how the emotional qualities of a stimulus affect many aspects of episodic memory, including the accuracy, vividness, consolidation, retrieval, and distortion of memories. A dominant approach to studying the effects of emotion on memory is to assess participants’ memory for stimuli that are emotionally evocative (e.g., snakes, spiders, guns) in terms of their affective qualities. The term “affect” is often used to describe various feeling states—including emotions, feelings, moods, motivations, and attitudes—that are experienced as having qualities of valence (i.e., pleasantness vs. unpleasantness) and arousal (i.e., alertness or activation; Barrett & Bliss-Moreau, 2009). In this article we build on these important foundations in memory research by applying a new set of ideas drawn from theories within affective science.

Affective science is an emerging field dedicated to understanding the mechanisms and consequences of affective states (Dukes et al., 2021; Gross & Barrett, 2013; Schiller et al., 2024). We argue that memory research could benefit greatly by considering the implications of psychological-constructionist theories from affective science, a family of mechanistic-emotion theories that offer an explanation of what emotions are and how they affect cognition and behavior. We use the tenets of psychological constructionism to advance a new model of emotional memory, which we call the *constructionist model of emotional memory* (CMEM). This model is compatible with many existing models of emotional memory, but it generates several new hypotheses and avenues of investigation for researchers interested in the intersection of emotion and memory.

## A (Necessarily) Brief Overview of the Extensive Literature on Emotion and Memory

Research has long demonstrated that the emotions one experiences during an event affect later memory (for reviews, see Kensinger & Ford, 2020; Kensinger & Schacter, 2016; Talmi, 2013). An example of early work in this area examined how individuals remembered highly emotional historic events, such as the assassination of U.S. President John F. Kennedy (Brown & Kulik, 1977; for more recent examples of such research, see Daley et al., 2025; Devarayapuram Ramakrishnan et al., 2024; Murphy et al., 2019). Since this early work, multiple theories have been devised to explain when and why people encode and remember emotional information differently than nonemotional information (Bowen et al., 2018; Mather & Sutherland, 2011; Talmi, 2013; Yonelinas & Ritchey, 2015). Our primary focus here is to articulate novel predictions generated by the CMEM. Thus, a comprehensive review of the extensive literature examining memory and emotion is beyond the scope of the current article. Nonetheless, we will briefly review some foundational findings to situate our model within the context of prior research.

The most common approach to studying emotion-memory interactions in the laboratory has been to investigate the mnemonic effects of two-dimensional features that characterize emotions: *valence* (or the extent to which a stimulus evokes positive vs. negative feelings) and *arousal* (or the extent to which a stimulus evokes feelings of activation vs. deactivation). Such research draws, whether implicitly or explicitly, upon Russell’s *circumplex model of affect*, which describes various emotional states as being accompanied by an orthogonal combination of valence and arousal (Russell, 1980).^1^ The most ubiquitous finding in emotional-memory research is that stimuli that evoke states high in positive or negative valence and high in arousal (i.e., that are emotional) are remembered better than those that do not (Buchanan, 2007; Dolcos et al., 2017; Kensinger & Ford, 2020; Kensinger & Schacter, 2016; Levine & Edelstein, 2009; Levine & Pizarro, 2004; Talmi, 2013). That is, emotional stimuli are typically better remembered than neutral stimuli (although, as discussed below, this depends on which aspects of memory are tested). In a classic example of emotionally enhanced memory, Kensinger and Corkin (2003) presented participants with emotionally negative words (e.g., *slaughter*) and neutral words (e.g., *brick*) and found that participants were better able to remember negative than neutral words (Experiment 1). The emotional enhancement of memory has been shown to generalize across different types of stimuli, with emotion benefiting memory for words (e.g., Kensinger & Corkin, 2003), narratives (e.g., Cahill & McGaugh, 1995), and images (e.g., Bradley et al., 1992; Cahill & McGaugh, 1995; Davidson & Vanegas, 2015). In addition to being better remembered, emotional stimuli are also typically remembered with greater vividness, confidence, and recollection (Bowen et al., 2018; Buchanan, 2007; Kensinger & Corkin, 2003; Kensinger & Ford, 2020; Kensinger & Schacter, 2016; Levine & Pizarro, 2004; Ochsner, 2000; Phelps & Sharot, 2008; Talarico & Rubin, 2003; Talmi, 2013). For example, across a series of experiments Ochsner (2000) found that emotional stimuli (and in particular, negative stimuli) were remembered with a greater sense of recollection than neutral stimuli.

At the cognitive level of analysis, emotionally enhanced memory is thought to be explained by differences between emotional and neutral information in terms of attentional allocation, as well as the degree of distinctiveness and interrelatedness with which such stimuli are represented (Talmi, 2013) At the neural level, emotionally enhanced memory has been related to the beneficial effects of amygdala-mediated consolidation of emotional-memory traces within the hippocampus (McGaugh, 2004). The enhanced sense of recollection when remembering emotional information is similarly thought to be due to the amygdala-mediated strengthening of memory representations for emotionally arousing information (Phelps & Sharot, 2008). The conclusion that emotion, in general, is beneficial to subsequent memory has been expanded upon by research demonstrating that valence and arousal lead to memory enhancements through distinct behavioral and neural mechanisms, with valence leading to memory enhancement through more controlled, prefrontal processes and arousal leading to memory enhancement through more automatic, amygdala-mediated processes (Kensinger & Corkin, 2004).

Although emotion tends to enhance memory, it does not do so for all aspects of an event. Instead, research suggests that, because emotional stimuli act as “attention magnets” (Laney et al., 2004) and are prioritized with respect to information processing (Mather & Sutherland, 2011), emotion tends to enhance memory for central, emotion-inducing details at the expense of the peripheral details of an episode (Dolcos et al., 2017; Kensinger, 2009a, 2009b; Kensinger et al., 2007; Levine & Edelstein, 2009; Levine & Pizarro, 2004; Mather & Sutherland, 2011). Kensinger et al. (2007) demonstrated this *memory trade-off effect* in an experiment in which participants studied negative and neutral images superimposed upon neutral background scenes. As with the emotionally enhanced memory effect, participants were more likely to remember the central images if they were emotional. Critically, however, participants were less likely to remember neutral backgrounds if they had been previously paired with an emotional image.^2^

### The nuanced effects of valence and arousal on memory

Despite the generalizations made in the above section, which were necessarily brief, it is important to acknowledge that investigations of the mnemonic effects of valence and arousal have given rise to a rich and nuanced line of scientific inquiry, as exemplified by the many theoretical perspectives that exist in this space (Bowen et al., 2018; Mather & Sutherland, 2011; Talmi, 2013; Yonelinas & Ritchey, 2015). Accounts such as Bowen et al.’s NEVER model (*negative emotional valence enhances recapitulation*; Bowen et al., 2018), for example, argue that rather than having identical effects on memory, positive and negative stimuli of comparable arousal may impact certain stages of memory differently, highlighting the possibility that important valence asymmetries may exist depending on which aspects of memory are tested (for additional discussions of possible valence asymmetries, see Kensinger, 2009a; Kensinger & Ford, 2020; Kensinger & Schacter, 2016; Larson & Steuer, 2009; for additional discussion of how emotion affects memory consolidation and retrieval, see Kensinger & Ford, 2020; Ritchey et al., 2008, 2013; Yonelinas & Ritchey, 2015). Additionally, and as alluded to earlier in our discussion of memory trade-off effects, perspectives such as Mather et al.’s *arousal-biased competition theory* emphasize that emotional arousal does not always result in a straightforward enhancement of memory but instead enhances the perception and memory of some event details at the expense of others (Mather & Sutherland, 2011), and that under certain testing conditions (e.g., when viewing degraded line drawings of previously seen images), may even result in worse memory for emotional relative to neutral objects (Faul et al., 2025). As yet another example, recent research has expanded on past findings of emotionally enhanced vividness by demonstrating that emotion has dissociable effects on the reconstruction of visual properties such as color, contrast, and hue (Faul et al., 2025). Furthermore, there are almost certainly exceptions to the generalizations made in the preceding section of our review; the effects of valence and arousal outlined above may not always co-occur (e.g., in some contexts, affect-related memory effects may be uncorrelated with affect-related attention effects; Monkman et al., 2025). Thus, the goal of our model is to generate novel research questions by drawing on ideas from affective science that are complimentary and orthogonal to existing ideas within this literature; we do not aim to encompass or replace the many nuanced perspectives within the field of emotional memory that have been invaluable to scientific progress within this area. Although it is clear that understanding the mnemonic effects of valence and arousal is essential to understanding emotional memory, we argue that this approach can be supplemented by drawing upon theories from affective science that provide mechanistic accounts of why valence and arousal, as well as categorical emotion states, might influence memory. In this regard, the CMEM is particularly novel in its predictions regarding the mnemonic effects of discrete emotions such as anger, disgust, and fear.

### Existing theoretical accounts of discrete emotion effects

In addition to the studies referenced here on the effects of valence and arousal on episodic memory, a smaller number of studies have investigated the mnemonic effects of specific discrete emotions (e.g., fear). The term *discrete emotions* is often used to refer to affective states that are experienced or perceived as bounded in time and place; linked to specific eliciting events; associated with specific types of meaning-making, and perhaps behaviors; and sometimes included in English-language categories such as “fear” or “afraid” (see Hoemann et al., 2025; Lindquist, 2013; Russell, 2003). For example, Chapman et al. (2013) found that participants had better memory for normatively disgusting images compared with normatively frightening images on tests of both recall and recognition. This finding has since been documented by others and termed the *disgust advantage* (Chapman, 2018; Chapman et al., 2013; Ferré et al., 2018; Marchewka, Wypych, Michałowski, et al., 2016; Moeck et al., 2021; Riegel et al., 2022; West & Mulligan, 2021, Experiment 3). Others have found that experiencing sadness is associated with increased memory for situational outcomes (Levine & Burgess, 1997; Levine & Edelstein, 2009; Levine & Pizarro, 2004), whereas experiencing anger is associated with increased memory for agents’ goals when memory is tested for narratives (Levine & Burgess, 1997). Thus, in addition to emotion affecting memory through valence and arousal, there is some evidence that discrete emotions affect memory as well.

The disgust advantage has largely been interpreted from the perspective of *basic-emotion theories*, according to which specific discrete emotions (e.g., fear, anger, disgust, etc.) represent distinct patterns of coordinated behavioral and physiological response in reaction to events within the environment (Ekman, 1999; Ekman & Cordaro, 2011; Tracy, 2014). It is important to note that these emotions are claimed to be basic in that they are thought to have evolved (via strictly genetic transmission during brain evolution; see Lindquist et al., 2022) to allow our ancestors to respond to recurring environmental challenges in adaptive ways. Furthermore, basic emotions are said to be universal across human cultures and are thought to be associated with unique physiological and behavioral response patterns. Although there is disagreement about which specific emotions are considered basic (Ortony & Turner, 1990), the ones most commonly agreed upon are fear, disgust, anger, sadness, happiness, and (sometimes) surprise (Ekman & Cordaro, 2011; Lindquist et al., 2012; Siegel et al., 2018).

Explanations of the disgust advantage draw on basic-emotion theories in that such explanations propose that disgusting and frightening stimuli differ in their memorability because of differences in the evolutionary functions served by these emotions. For example, Chapman (2018) theorized that disgusting information may be particularly memorable because, relative to physical threats that evoke fear, disease-related (i.e., disgusting) threats may be more subtle in nature and may require additional attention to evaluate. This additional attention at encoding, it is reasoned, may explain why disgusting stimuli are ultimately better remembered than frightening stimuli (but see Chapman et al., 2013; Moeck et al., 2021). Similarly, neuroimaging researchers have drawn upon basic-emotion theories when interpreting the neural correlates of disgust-related memory (Marchewka, Wypych, Michałowski, et al., 2016; but see Marchewka, Wypych, Moslehi, et al., 2016).

Causal-appraisal theories of emotion have also been used to interpret the effects of discrete emotions on memory (Kaplan et al., 2012, 2016; Levine & Burgess, 1997; Levine & Edelstein, 2009; Levine & Pizarro, 2004). Briefly, causal-appraisal theories posit that emotions result from one’s interpretations (i.e., appraisals) of the current situation, with specific patterns of appraisals resulting in specific discrete emotions, which in turn have specific and consistent effects on behavior and physiology (Roseman & Smith, 2001).^3^ For example, according to causal-appraisal theories, sadness results from the appraisal that something important has been lost and encourages passivity, whereas fear is thought to result from the appraisal that the current situation is dangerous and encourages avoidance (Moors, 2014; Roseman & Smith, 2001). Memory researchers adopting a causal-appraisal perspective have argued that discrete emotions and their corresponding appraisals determine which aspects of an event are goal congruent and ought to be prioritized by memory (for reviews, see Levine & Edelstein, 2009; Levine & Pizarro, 2004).

Notably, memory researchers adopting basic-emotion or causal-appraisal perspectives interpret discrete emotion effects within a given experiment as evidence that such emotions affect memory in specific and consistent ways across instances. That is, whether finding differences between discrete emotions in terms of memorability (Chapman, 2018; Chapman et al., 2013; Ferré et al., 2018; Marchewka, Wypych, Michałowski, et al., 2016; Moeck et al., 2021; Riegel et al., 2022; West & Mulligan, 2021, Experiment 3), the neural correlates of memory (Marchewka, Wypych, Michałowski, et al., 2016), or their effects on the aspects of a narrative that are remembered (for reviews, see Levine & Edelstein, 2009; Levine & Pizarro, 2004), such differences are interpreted as the effects of these specific emotions, resulting from preprogrammed patterns of context-free cognition and relationships between a given emotion and memory that are one to one. Variability in these models—whether across individuals or contexts—is seen as error rather than an interesting effect to be explained. Such interpretations stand in stark contrast to those that follow from constructionist theories of emotion.

As reviewed above, prior emotional-memory research has largely taken one of two approaches to investigating the effects of emotion on memory, when the effects of affective features such as valence and arousal or the effects of discrete emotions have been considered. Often, perspectives applied to the emotional-memory literature focus on the role of valence and arousal or discrete emotions, but not to consider both in tandem. With this in mind, the consideration of well-developed theories from affective science that provide mechanistic accounts of both affect and discrete emotion may be of great benefit to emotional-memory researchers by facilitating the generation of novel, theoretically informed research programs. In the current article, we consider the utility of one class of emotion theories that has received considerable support in recent years: psychological constructionism (Barrett, 2014, 2017a, 2017b; Lindquist, 2013), which sees emotions as emergent phenomena produced by more basic psychological and neural mechanisms that are highly idiographic and situated in context and culture.

## Psychological Constructionism: A Theoretical Primer

Psychological-constructionist theories of emotion posit that emotions are contextually sensitive, emergent phenomena that arise as a result of the brain’s attempts to make meaning of sensations in the world around us and within our bodies using conceptual knowledge learned through prior autobiographical experiences, social learning, or both (Barrett, 2014, 2017a, 2017b; Barrett et al., 2025; Cunningham et al., 2013; Lindquist, 2013; Satpute et al., 2020). Constructionist theories conceive of emotions as “constructed” in that emotions are thought to result from the interaction of more basic psychological features, such as external sensory information, internal affective sensations, and conceptual knowledge about the emotion concepts most relevant to one’s culture. Although there are a number of constructionist theories that are united in these core tenets (e.g., Barrett, 2013; Clore & Ortony, 2013; Coan, 2010a, 2010b; Lindquist, 2013; Lindquist et al., 2022; Russell, 2003, 2009), in the current article we focus on Barrett and colleague’s *theory of constructed emotion* (Barrett, 2017a, 2017b; previously referred to as the *conceptual act model*, Barrett, 2014; Barrett et al., 2015).

The theory of constructed emotion assumes that a primary purpose of the brain is to guide an organism’s actions in such a way that the organism is able to cope with ongoing physiological demands within the environment (Barrett, 2017a, 2017b). To accomplish this, the brain generates and maintains an ongoing internal model (i.e., a simulation) of the body within the context of the external world (Barrett & Simmons, 2015; Kleckner et al., 2017). This simulation is thought to be made possible by predictive processing, wherein the brain continuously generates and tests competing predictions regarding the meaning of current external and internal sensations. This is done on the basis of prior experiences and conceptual knowledge, and the brain updates those predictions in the face of incoming sensory information from the world and the body (Barrett, 2017b; Friston, 2010; Seth & Friston, 2016). According to the theory of constructed emotion, emotions are constructed and experienced as a product of such predictive processing.

One psychological feature necessary for the construction of emotion is *affect*, or the brain’s representation of the state of the body in terms of valence and arousal (Barrett, 2005; Barrett & Bliss-Moreau, 2009; Feldman et al., 2024; Russell, 2009). Expectations regarding the visceromotor impact of external stimuli are predicted to result in the experience of these lower-order affective sensations represented via interoception (Barrett, 2017b; Barrett & Bliss-Moreau, 2009; Shaffer et al., 2023). The experience of valence, for example, is thought to result from the prediction that a stimulus might have beneficial (positively valenced) or harmful (negatively valenced) effects on oneself (Barrett, 2006). Valence also appears to be affected by confidence in one’s predictive model (Hesp et al., 2021). Arousal, on the other hand, has been theorized to result from the predicted need for action in response to a stimulus (e.g., the predicted need to run away from a snake), or the need to pay further attention to update one’s predictive model (Barrett, 2006, 2017b; Feldman et al., 2024). Russell (2003) referred to the process wherein sensations of valence and arousal are attributed to external stimuli as the *perception of affective quality* (which we refer to as “the perception of affect” throughout the remainder of the article), a process that we expand upon further below.

Although necessary for emotion, the theory of constructed emotion predicts that affect alone is insufficient for discrete emotional experience. Indeed, according to Barrett (2015), constructionist theories are sometime mistakenly thought to be dimensional theories, which reduce emotions entirely to the dimensions of valence and arousal. Because the theory of constructed emotion conceives of categorical emotion states as emergent phenomena that cannot be reduced to affective sensations alone, the theory of constructed emotion is not a dimensional theory and therefore acknowledges the psychological reality of discrete emotions.

Notably, it is the combination of affect with categorization^4^ that constructionist theories hypothesize gives rise to discrete experiences of emotion. *Categorization* refers to the process by which one uses prior knowledge to predict that the external sensory sensations and the internal affective sensations constitute an instance of a specific emotion category. This prior knowledge can be thought of as a set of emotion concepts (e.g., fear, sadness, anger; Barrett, 2005, 2014, 2017a, 2017b; Brosch et al., 2010; Lindquist, 2013; Wilson-Mendenhall et al., 2011) that consist of populations of variable category instances encoded in semantic memory. Such instances vary in their features (Barrett, 2009), even within a category (Barrett, 2017a). Such variation can be a product of situational factors (fear of heights has different features than fear of spiders and fear of social encounters; e.g., McVeigh et al., 2024; Wang et al., 2024); individual differences in experience and learning (see Hoemann, Nielson, et al., 2021); and cultural differences in values, norms, and socialization practices associated with emotions (see Lindquist et al., 2022). The primary thesis of the theory of constructed emotion is thus that emotions are constructed when one uses learned emotion concepts to categorize and interpret external sensory and internal affective sensations within a given situational context (Barrett, 2014, 2017a, 2017b; Lindquist, 2013; Wilson-Mendenhall et al., 2011). For the remainder of the article, we will refer to this process as *emotion construction*.

The processes involved in the perception of affect and emotion construction have several consequences worth expanding upon. With regard to the former, it should first be noted that affective sensations of valence and arousal are thought to be constant aspects of conscious experience (Barrett & Bliss-Moreau, 2009) and are sometimes experienced as free-floating and nonspecific when not perceived as qualities of a specific stimulus within the environment (Barrett, 2005; Russell, 2003). On occasions when a stimulus is seen as the perceived cause of changes in affect, affect is experienced as being “about” the stimulus (Barrett, 2005, 2006, 2017a; Barrett et al., 2015; Russell, 2003); that is, affective sensations become *attributed affect* (Russell, 2003). It is important to note that this perception of affect may occur with or without emotion construction (Bliss-Moreau, 2017; Lindquist, 2013) and likely forms the basis of states colloquially called “moods,” “attitudes,” or “feelings” toward particular stimuli. In the absence of emotion construction, stimuli associated with attributed affect may be perceived as having a particular degree of valence and arousal but may not be associated with a particular discrete emotion. The perception of affect in the absence of categorization may occur because of the development of “experiential habits” (Barrett et al., 2015), wherein certain affective sensations become associated with a given stimulus following (a) standard principles of associative learning (Lindquist, 2013), (b) the experience of concurrent shifts in affect originating from causes external to the stimuli (e.g., increases in arousal due to the ingestion of caffeine; Lindquist, 2013), or (c) disruptions of or failures to engage in emotion construction (see Prediction 2 below for an extensive discussion of this last point).

On other occasions, affective sensations may be categorized as experiences of discrete emotions when the perceived object is infused with not only valence and arousal, but also conceptual features of the associated emotion, rendering that stimulus not only affective but also emotional to the perceiver (Barrett, 2005, 2006, 2017a; Barrett et al., 2015; Russell, 2003). As a case in point, a stimulus that is categorized as fearful is not only predicted to be negative, arousing, and aversive, but might also invoke predictions about its threat, certainty, and controllability, and about specific actions a person might undertake in that context (Brosch et al., 2010; Duncan & Barrett, 2007; Lindquist & Barrett, 2008b).

To illustrate the process of emotion construction, consider, for example, a situation in which you cross paths with a brown object lying horizontally in your path while taking a hike. If you generate a prediction that this object is a stick, there may be no emotional consequences: a stick is expected in the woods, and provided it is not a tripping hazard, it has no consequences for you. The result is that the object would be perceived as being neutral in valence and not highly arousing. However, it is also possible as you approach the stick that your brain cannot resolve what this object is. This failure to predict the meaning of the object might result in a feeling of both arousal and negative valence and would encourage further visual attention to resolve the meaning of the object. This would be an instance in which the stimulus has affective, but not necessarily discrete, emotional qualities. In common-sense experience, the feelings of arousal and valence are said to emanate from the object, but constructionist theories suggest that these are predicted sensations resulting from the brain’s unresolved and resolved meaning-making of visual sensations of the object. At the same time, it is possible that if you are hiking in an area where you might expect to encounter a snake, your brain might predict that this object is a snake. Provided that you associate snakes with fear, this specific prediction would result in a categorical experience of fear characterized by visceromotor plans to avoid the snake, resulting in arousal as well as the valenced feelings of unpleasantness associated with the potential consequences of being bitten. Again, in common-sense experience, the snake is said to be threatening or to cause fear. However, from a constructionist perspective, fear is a predicted consequence of the stimulus situated within the perceiver. However, there may be exceptions to this general example; for a herpetologist out seeking new specimens, the prediction that a brown object in their path is an Eastern diamondback rattlesnake might not generate fear, but rather pleasantly valenced states, such as interest or excitement. What remains in question is whether the brown object is remembered better if predicted to be neutral rather than negative and highly arousing rather than fearful. This is among the types of questions generated by a constructionist account of memory.

## A Constructionist Account of Discrete Emotions and Their Effects on Cognition

Having provided an overview of the theory of constructed emotion, we will now discuss how this theory conceives of discrete emotions and their effects on cognition. Although the theory of constructed emotion takes into account the reality of categorical emotional experience, this theory does not conceive of discrete emotions (e.g., disgust, fear) as having one-to-one relationships with consistent and specific patterns of cognitive, behavioral, or physiological response (Barrett et al., 2015), because different instances of the same emotion are predicted to vary considerably from one another on the basis of contextual factors. Instead, a given instance of a discrete emotion such as fear is only one possible instance drawn from a population of variable fears, and it is constructed on the basis of the unique features associated with a given context (Barrett, 2015, 2017a). Consequently, the specific patterns of cognition, behavior, and physiology associated with an instance of a specific emotion (e.g., fear) are predicted to vary depending on the response that is predicted to be optimal for an individual within a given situation, based on that individual’s prior experiences (Barrett, 2015; Barrett et al., 2015).

Whereas basic-emotion and causal-appraisal theories predict that certain discrete emotions will have singular effects on cognitive processes such as memory, the theory of constructed emotion predicts that the effects of discrete emotions on memory will vary considerably between situations, individuals, and cultures (Barrett, 2009). Indeed, growing evidence suggests that peripheral physiological responding (Hoemann, et al., 2020; McVeigh et al., 2024), brain representations (Doyle et al., 2022; Wang et al., 2024), perceptions of emotion in facial behaviors (Brooks & Freeman, 2018), and even emotion-concept meanings (Jackson et al., 2019) vary considerably within instances of an emotion category such as fear and are predicted by the situation, the individual, the culture, and variation in the specific concept knowledge the person brings to bear.

In this way, constructionism addresses an important limitation of research on the mnemonic effects of discrete emotions as informed by basic-emotion and causal-appraisal theories, namely that, by asserting that specific emotions affect memory in singular ways, such research is unable to account for potential sources of variability that occur between instances of a given emotion category. Given evidence of within-emotion heterogeneity in terms of experience and appraisal (Hoemann et al., 2020; Nezlek et al., 2008), facial perception and expression (Barrett et al., 2019), autonomic physiology (Siegel et al., 2018), and neural activation (Clark-Polner et al., 2016; Lindquist et al., 2012),^5^ there is a pressing need for memory researchers to consider alternative theories from affective science that are able to account for the substantial variability that exists in emotional experience.

By appreciating the psychological reality of discrete emotions while also emphasizing the important role that sources of variability in emotion construction will have on discrete emotions, the theory of constructed emotion is able to accommodate discrete emotion effects, such as those described above, as consequences of contextually sensitive emotion construction while also generating valuable hypotheses about the boundary conditions of such effects that do not follow from alternative emotion theories, some of which are reviewed below. After articulating the CMEM and its predictions, we will return to these points to interpret existing effects of discrete emotions found in the memory literature.

## The Constructionist Model of Emotional Memory

An advantage of constructionist theories as applied to research on emotion and cognition is that, because these theories incorporate domain-general cognitive phenomena—such as predictive coding (Barrett, 2017b; Friston, 2010; Seth & Friston, 2016); associative learning (Barrett et al., 2015; Lindquist, 2013); perception and interoception (Barrett & Simmons, 2015; Brosch et al., 2010; Fugate et al., 2010; Kleckner et al., 2017); and the organization, availability, and accessibility of conceptual knowledge (Barsalou, 2003, 2008, 2009) as central to the construction of emotion—this perspective naturally integrates with approaches addressing cognitive phenomena such as memory, and with cognitive science more broadly. Constructionist perspectives have informed theorizing regarding the relationship between emotion and cognitive phenomena such as language (Barrett et al., 2007; Fugate & Barrett, 2014; Lindquist, 2017; Lindquist & Gendron, 2013; Lindquist, MacCormack, & Shablack, 2015; Lindquist, Satpute, & Gendron, 2015; Lindquist et al., 2016) and visual perception (Barrett, 2017a; Barrett & Bar, 2009; Barrett et al., 2011; Brosch et al., 2010; Gendron, 2017; Gendron & Barrett, 2018; Gendron et al., 2012; Kveraga et al., 2015; Wilson-Mendenhall et al., 2011), but the implications of the theory of constructed emotion have yet to be considered with regard to the role of emotion in episodic memory. It is our primary goal in this article to address this gap. To this end, we articulate a model of emotional memory rooted in constructionism: the CMEM.

**Fig. 1.**
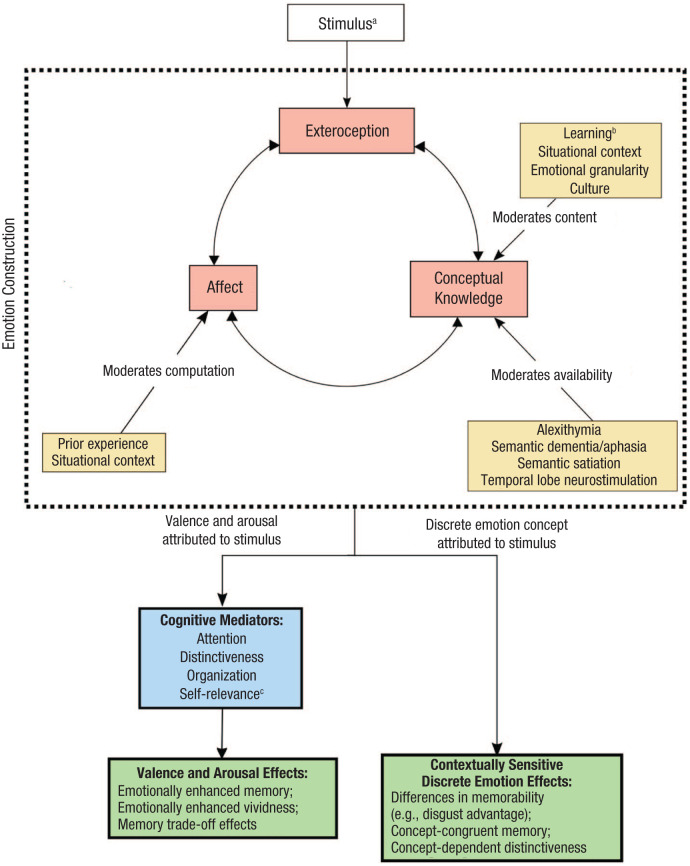
The constructionist model of emotional memory. The effects of emotion on memory are explained by the attribution of affective sensations and discrete emotion concepts to a stimulus through emotion construction, which in turn depends on external perceptual inputs (i.e., exteroception), affective sensations, and conceptual knowledge, as well as the factors that influence these processes. Predicted moderators are depicted using brown boxes, though the list of moderators is selective rather than exhaustive. Note that our use of single-headed arrows in this model is not meant to imply that the depicted effects are linear and nonrecursive but is rather a visual simplification meant to aid in hypothesis generation. ^a^The word “stimulus” is used in an intentionally broad manner and may refer to anything that might be perceived as emotional (e.g., images, words, faces, people, events). ^b^Likewise, the word “learning” is used in a broad sense and refers to prior experience, development, and acculturation. ^c^“Self-relevance” is included but marked for its relatively untested status as a mediator of the effects of valence and arousal on memory.

The CMEM is presented in Figure 1. It is important to note that, because the theory of constructed emotion conceives of emotion construction as a dynamic and recursive process (Barrett, 2015), this model is not meant to imply that the effects of emotion on memory proceed in a linear, nonrecursive manner; instead, the process is simplified for the purpose of hypothesis generation. It would not be surprising if future research were to identify recursive or bidirectional relationships not explicitly depicted in Figure 1. Similarly, we note that because processes such as affective sensation and categorization are thought to be fundamental aspects of consciousness, one’s mind is never a blank slate waiting to be perturbed by an emotional stimulus (Barrett, 2015). Said differently, a perceiver always has a prior mental state, which will inevitably influence how a stimulus is experienced and ultimately remembered.

A central thesis of the CMEM is that many effects of emotion on memory are determined by the pattern of information processing that results from emotion construction as described by the theory of constructed emotion (Barrett, 2017a, 2017b). We provide an explanation of how the CMEM accounts for the effects of discrete emotions on memory toward the end of the article after we have had the chance to discuss constructionist research related to discrete emotions more fully. Regarding the typical effects of valence and arousal on memory, the CMEM proposes that these effects are dependent on the attribution of internal affective sensations to external stimuli preceding and during emotion construction. Following emotion construction, the stimulus will then be perceived as being the source of these feelings and will be seen as emotional. Following the attribution of affective sensations to a stimulus, we assume that the cognitive mediators described by Talmi’s mediation theory (2013) account for the typical effects of valence and arousal on memory (see Fig. 1). In other words, we assume that affect or emotion will result in better, more vivid memories and in memory trade-offs as a result of emotional information receiving increased attentional allocation (for a review, see Carretié, 2014), being seen as highly distinctive (i.e., standing out; Talmi, 2013; Talmi & McGarry, 2012; Talmi et al., 2007), and being seen as more conceptually interrelated than neutral stimuli (Talmi, 2013; Talmi & Moscovitch, 2004; Talmi et al., 2007). The tendency for emotional stimuli to attract more attention and to be seen as more distinctive than neutral stimuli is predicted to be due to the perception of emotional stimuli as being salient and consequential to one’s well-being (Barrett, 2006; Brosch et al., 2010; Duncan & Barrett, 2007). The tendency to perceive emotional stimuli as being more interrelated than neutral stimuli is predicted to be due to the perception of emotional stimuli as belonging to common conceptual categories such as “emotional,” “good,” and “bad” (Lindquist & Barrett, 2008b).

In addition to the cognitive mediators of attention, distinctiveness, and organization proposed by Talmi’s mediation theory (2013), we propose that the mnemonic effects of valence and arousal might also be explained by the increased self-relevance of emotional stimuli. Stimuli perceived as valenced and arousing are likely inherently more self-relevant than neutral stimuli insofar as affect is thought to signify a stimulus’s impact for the body (Barrett, 2017a; Feldman et al., 2024; Shaffer et al., 2022). Yet the prediction that a stimulus has discrete emotional meaning likely intensifies its self-relevance above and beyond valence and arousal. After all, emotion concepts name states that confer meaning about how a stimulus situation impacts the individual (Hoemann et al., 2025). The notion that emotional stimuli are self-relevant is an important aspect of constructionist theorizing. For example, Barrett (2017a) stated that any stimulus that is predicted to have a significant impact on one’s well-being (e.g., an emotional stimulus) will be seen as personally meaningful (see also Duncan & Barrett, 2007). Furthermore, as noted earlier, there may be recursive relationships between emotion construction and cognitive mediators such as self-relevance. Suggestive of this possibility is the finding that judgments of self-relevance enhance the emotional intensity of considered events (e.g., Jeunehomme & D’Argembeau, 2017; see also Stendardi et al., 2021; Thomsen & Pillemer, 2017). Classic appraisal dimensions describe ways in which an emotional stimulus is or is not relevant to the self (e.g., whether it was caused by the self or other, whether it is relevant and congruent with one’s goals, whether it is controllable; Ellsworth & Klaus, 2003). That is, the emotionality of a stimulus or an event may affect the extent to which it is perceived as being self-relevant, and, in turn, considerations of self-relevance may influence the degree of emotional scrutiny applied to the stimulus or event, influencing emotional intensity.

Recent research has pointed to overlap between the mnemonic effects of emotion and self-relevance. For example, Gutchess and Kensinger (2018) reviewed evidence that emotional and self-referential stimuli influence memory through similar mechanisms (but see Daley et al., 2020), and argued that the mnemonic effects of emotion and self-referential processing should be integrated into a shared model. Research on the self-reference effect in memory has amply demonstrated its interaction with emotional attributes of stimuli, supporting this view (e.g., Liu et al., 2024; Moses-Payne et al., 2022; Szpunar et al., 2011). Because some have theorized that emotional stimuli are experienced as personally relevant, and because some research has demonstrated overlap between the mnemonic effects of emotion and self-referential processing, we propose that—as with attention, distinctiveness, and organization—self-referential processing may partially explain the mnemonic effects of valence and arousal and perhaps, to a greater extent, discrete emotion effects. It is our hope that this claim will inspire further research within this area of investigation.

With regard to the mnemonic effects of valence and arousal, the CMEM can be seen as providing additional value to Talmi’s mediation theory (2013) in that our model contextualizes mediation theory’s explanations of emotional-memory effects within the mechanistic explanation of emotion presented by the theory of constructed emotion. Furthermore, by placing emphasis on the processes that influence how valence and arousal come to be attributed to stimuli in the first place, the CMEM is able to generate specific predictions regarding the conditions under which the typical effects of valence and arousal on memory may or may not manifest, some of which we review below.

Perhaps the most exciting aspect of the CMEM is its novel predictions regarding the effects of emotion construction on memory. Two sorts of predictions follow from the CMEM, as depicted in Figure 1. First, our model predicts that factors central to emotion construction, such as conceptual knowledge, will affect later memory. Second, given that our model predicts that the effects of emotion on memory depend on the products of emotion construction, it follows that any factor that affects emotion construction will influence how stimuli are ultimately remembered. In the following sections we discuss the implications of these assertions, articulated as a series of predictions. More specifically, we discuss predictions regarding the biasing effects of discrete emotion concepts on memory, the role of conceptual knowledge in emotional-memory effects, and the effects of variability in emotion construction on memory. A summary of these predictions and their current evidentiary status is presented in Table 1. Rather than representing an exhaustive list of every implication the CMEM has for emotional-memory research, these predictions are intended to function as a starting point meant to inspire further research. Here we focus on what we see as the most fruitful first steps in evaluating the CMEM.

**Table 1.** Emotional Memory Hypotheses Generated by the Constructionist Model of Emotional Memory and their Current Evidentiary Status

Constructionist premise	Hypothesis about emotional memory	Basis for hypothesis	Evidentiary status	Direct evidence	Other relevant findings from prior research
Emotion construction entails the categorization of affective sensations using learned emotion concepts.	The categorization of stimuli using discrete emotion concepts will bias later memory in a concept-congruent manner.	Theory of constructed emotion (TCE)	Direct	Providing participants with emotion labels for facial expressions results in emotion-congruent memory biases.	Providing semantic categories for nonemotional stimuli results in concept-congruent memory biases.
Conceptual emotion knowledge is necessary for emotion construction, and emotion construction is necessary for the experience of discrete emotion.	When access to conceptual emotion knowledge is reduced, the effects of discrete emotions on memory will be reduced. Such effects include (a) concept-congruent memory biases, (b) concept-dependent distinctiveness, and (c) discrete emotion effects, such as the disgust advantage.	TCE	Untested	N/A	Individuals with sematic dementia have deficits in discrete emotion perception. Semantic satiation disrupts discrete emotion perception. Acquiring conceptual knowledge results in the enhancement of between-category differences in perception.
Conceptual emotion knowledge is necessary for emotion construction, and emotion construction may increase the specificity and intensity of valence and arousal.	When access to conceptual emotion knowledge is reduced, the typical effects of valence and arousal on memory will be reduced.	TCE	Direct	Individuals with alexithymia show reduced emotionally enhanced memory and recollection. Patients with semantic dementia show disruptions in emotional memory.	Individuals with alexithymia are less likely to view external stimuli as emotional and tend to somaticize their affective sensations. Patients with semantic dementia show disruptions in emotion perception and somaticize their affective sensations. Patients with semantic aphasia show disruptions in emotion perception.
Features of the current situation influence emotion construction.	Context-framing manipulations will affect emotional memory.	TCE	Direct	Framing nonemotional stimuli as having been touched by contaminated hands enhances memory.	Context-framing manipulations affect emotion/affect perception.
	The effects of a given emotion (e.g., fear) on memory will depend on the situation within which it is experienced.	TCE	Untested	n/a	Instances of the same emotion differ depending on the situation in which they are experienced.
There is substantial interindividual variability in emotion/affect perception.	Individual perceptions of emotion/affect will predict memory better than normative ratings.	TCE	Preliminary	Unpublished work suggests that idiosyncratic ratings of valence predict image recall better than normative ratings of valence for the same stimuli.	
There is substantial individual variability in emotion construction because of differences in emotional granularity.	Highly granular individuals will show increased emotional memory, particularly for materials that encourage nuanced emotion construction.	TCE, supplemented by prior emotion research	Preliminary	Unpublished work suggests that participants high in negative granularity show stronger effects of valence on image recall.	Highly granular individuals represent emotional stimuli and events in a more distinctive manner.
	Emotional granularity will be associated with individual differences in the neural correlates of emotional memory.	TCE, supplemented by prior emotion research	Untested	n/a	Emotional granularity is related to differences in the neural representation of emotional information as measured by electroencephalogram (EEG).
Emotion construction is influenced by emotion concepts that are situated within the context of culture.	Cultures will differ in whether participants show specific discrete emotion effects (e.g., the disgust advantage).	TCE	Untested	n/a	Unconstrained methods show cross-cultural variability in the perception of discrete emotions, and some languages do not have words for certain emotions.
	Cultures will differ in whether emotional memory effects are moderated by social context.	TCE, supplemented by prior emotion research	Untested	n/a	Individuals from some cultures are more likely to see situations as emotional if they are social. Individuals in some cultures are influenced by the emotions of task-irrelevant others during emotion perception.
	Specific emotions will exert stronger effects on memory in cultures where they are reinforced compared with cultures where they are not reinforced.	TCE, supplemented by prior emotion research	Untested	n/a	Cultures differ in which emotions are reinforced, and culturally reinforced emotions are experienced with greater prevalence and intensity.
	The extent to which relatedness mediates emotional-memory effects will differ between cultures.	TCE, supplemented by prior emotion research	Untested	n/a	Certain cultures appear to not possess an explicit concept of emotion and may therefore not conceptualize emotional stimuli as being related to the same degree as in cultures that do possess an explicit emotion concept.
Constructionist theories do not conceive of discrete emotions as having one-to-one effects on cognition, behavior, or physiology.	Discrete emotions will not have specific and consistent effects on emotional memory or its physiological correlates. Instead, discrete emotion effects will be contextually sensitive and will depend on situational features as well as differences in the nature and availability of conceptual emotion knowledge between people and cultures.	TCE	Untested	N/A	There is considerable variability in the behavioral and physiological responses associated with discrete emotions. Discrete emotion construction and perception varies between situations, individuals, and cultures.

Note: Hypotheses are differentiated in terms of whether they follow directly from the first principles of the theory of constructed emotion or whether they are motived by a combination of the theory of constructed emotion (TCE) and additional emotion research that supplements this theory. For instance, many of our predictions regarding cross-cultural differences in emotional memory are motivated by TCE, supplemented by prior cross-cultural research that has clarified the specific ways that cultures differ in the conceptual knowledge that they transmit about emotions. Hypotheses are classified as having direct evidence if they are supported by prior emotional-memory research and as untested otherwise.

## Prediction 1: Emotion Concepts Will Bias Emotional Memory

A key assertion of constructionist theories is that emotions result from the categorization of external sensory information and internal affective sensations using learned emotion concepts (Barrett, 2014, 2017a, 2017b; Lindquist, 2013; Wilson-Mendenhall et al., 2011). The CMEM thus integrates the previously independent fields of research on emotional memory with research on the mnemonic effects of semantic categories (Carmichael et al., 1932; Hourihan et al., 2013; Hugenberg & Sacco, 2008; Lutz, 1983; Pauker et al., 2009). More specifically, the CMEM predicts that the emotion concepts drawn on during emotion construction will alter the way that emotional stimuli are later remembered in a concept-congruent manner, likely above and beyond the mere effects of valence and arousal. Consistent with this possibility, prior research demonstrates that access to an emotion concept, such as fear, changes emotional behavior (K. M. Lee et al., 2022; Lindquist & Barrett, 2008a; Oosterwijk et al., 2010) and experience (Lee et al., 2018) beyond the effects of negative valence and high-arousal affect alone.

Previous research has shown that accessing category information during the perception of ambiguous stimuli results in concept-congruent memory biases (Carmichael et al., 1932; but see Prentice, 1954). In a classic experiment, participants were presented with ambiguous line drawings alongside verbal descriptors (Carmichael et al., 1932). For example, a line drawing was presented with the text “curtains in a window” or “diamond in a rectangle.” When asked to reproduce these drawings from memory, participants tended to redraw the shapes in ways that exhibited features of the descriptions that they had previously been given. Semantic categories can also bias memories of more complex, socially relevant stimuli (for a review, see Hugenberg & Sacco, 2008). For example, Lutz (1983) found that participants integrated more heterosexual stereotypes when recalling the case history of a woman described as “heterosexual” compared with “homosexual.” Additionally, it has been shown that the accuracy with which racially ambiguous faces are remembered can be altered by providing disambiguating racial labels at encoding (Hourihan et al., 2013; Pauker et al., 2009).

Such research provides ample evidence that applying category information to ambiguous stimuli affects how they are remembered, a phenomenon we refer to as *concept-congruent memory*. To the extent that emotion construction accesses emotion concepts during the processing of otherwise ambiguous stimuli (Barrett, 2014, 2017a, 2017b; Lindquist, 2013; Wilson-Mendenhall et al., 2011), this categorization ought to affect memory in a similar manner—that is, concept-congruent memory ought to characterize emotional memory (see Fig. 1). More specifically, it should be the case that one’s memory of an emotional stimulus will be biased to exhibit features of the specific emotion concept accessed by a person during emotion construction.

Preliminary support already exists for concept-congruent memory as applied to emotional stimuli in research demonstrating that the presentation of emotion-category labels leads to concept-congruent biases in memory for facial expressions. For example, in a series of short-term memory experiments, Fugate et al. (2018) demonstrated that when primed with an emotion word, participants were biased toward falsely perceiving facial-muscle movements representative of that emotion. In regard to long-term memory, Halberstadt and colleagues conducted a series of studies in which participants were presented with emotionally ambiguous facial expressions and were asked to provide a verbal explanation as to why the target person might be experiencing a specific emotion (e.g., why they were feeling anger or happiness; J. Halberstadt, 2005; J. B. Halberstadt & Niedenthal, 2001; J. Halberstadt et al., 2009). When later asked to recognize faces along an expressive continuum, participants remembered faces as having been closer to stereotyped expressions of the described emotion than they were (for a more recent demonstration that emotion labels bias long-term memory for facial expressions in a concept-congruent manner, see McMullen et al., 2025). For example, participants who had previously explained why someone was feeling angry remembered that person’s expression as having been closer to a stereotypical expression of anger than it originally was (J. B. Halberstadt & Niedenthal, 2001). These findings suggest that it is not the perceived valence of facial-muscle movements alone that influences memory, but rather that accessing emotion concepts to make meaning of those facial-muscle movements further alters memory of the faces.

In another example of the biasing effects of emotion concepts on long-term memory, Doyle and Lindquist (2018, Experiments 1 and 2) experimentally manipulated participants’ knowledge of novel emotion concepts to better isolate the effects of emotion knowledge on later memory. In a learning phase, participants repeatedly viewed eight alien faces, created using 3D modeling, that displayed expressions of novel emotions that were labeled “blurp” and “gep.” Participants differed in terms of whether they were taught to classify faces using these emotion concepts or whether they classified faces on the basis of skin color. During the target phase, each alien was assigned to one of the two novel expressions, and participants were shown the aliens’ names along with multiple images of the aliens adopting subtly different variations of their respective expressions. Following a delay, participants completed a recognition memory test in which they were asked to select the expression that each alien had displayed during the target phase. Distractors for this test included the alien’s original expression from the learning phase and a morph that combined both expressions. Results showed that participants who had previously been taught novel emotion concepts were more likely to wrongly remember having seen the expression originally adopted during the concept-learning phase, suggesting that even artificial emotion concepts taught to participants in a single visit to the lab can induce concept-congruent memory biases.

Thus far, research investigating the biasing effects of emotion concepts on memory has exclusively investigated participants’ memory of emotional facial behaviors. The CMEM makes the novel prediction that such concept-congruent memory biases should generalize to all manner of stimuli, including emotionally charged objects and events. Furthermore, although the extant literature on the mnemonic influences of emotion concepts has taken an approach in which emotion words are provided by the experimenter and made salient to participants at encoding (J. Halberstadt, 2005; J. B. Halberstadt & Niedenthal, 2001; J. Halberstadt et al., 2009), the CMEM predicts that—because the access of emotion concepts is predicted to be a necessary aspect of emotion construction (Barrett, 2014, 2017a, 2017b; Lindquist, 2013; Wilson-Mendenhall et al., 2011)—the effects of emotion concepts on later memory should appear regardless of whether emotion concepts are provided by an experimenter or generated automatically and implicitly by participants (e.g., as a prediction based on the context at hand).

How might we expect emotion concepts accessed during emotion construction to shape our memories for emotional events? Broadly speaking, the CMEM predicts that people will tend to remember emotional events as having exhibited features that are conceptually associated with the specific emotion they felt during encoding. As an example, suppose that two individuals go to the hospital to receive stitches for a similar injury. Suppose, also, that these two individuals possess relatively comparable emotion concepts associated with fear and disgust, perhaps because these individuals were socialized within the same culture (for a further discussion of cultural influences on emotion construction, see below). Because of factors that differ between these individuals, they may access different emotion concepts to construct their emotions during this event. If they experience fear during the procedure, we might expect those individuals to remember this event as having had more features associated with their conceptualization of fear than it did. For instance, a particular individual may remember the doctor as being more malicious than he or she actually was, the outcomes of the treatment as more uncertain, or the doctor’s stitching needle as larger than it was. In contrast, another individual may experience the same procedure as disgusting. We might expect this person to remember the event as having exhibited features associated with a conceptualization of disgust which it did not exhibit—perhaps the medical station was remembered as being less clean than it was, or perhaps the injury was remembered as being bloodier than it was in reality.

Should future research support the prediction that the self-generated and automatic categorization of emotional stimuli and events biases how such information is remembered, it will be important to determine the mechanisms underlying this effect. It could be that the predicted effects of emotion concepts on later memory operate through a reconstructive process by which an emotion concept generated during emotion construction influences how an emotional event is reconstructed at retrieval. Insofar as emotion concepts might be conceptualized as knowledge structures similar to schemas,^6^ such an explanation would be consistent with reconstructive accounts of schema-congruent memory distortions (Bower et al., 1979; Brewer & Treyens, 1981; Hirt, 1990; Hirt et al., 1999). Alternatively, it may be that the mnemonic effects of emotion concepts operate by affecting encoding. J. Halberstadt (2003, 2005) and J. B. Halberstadt and Niedenthal (2001), for example, have argued that the emotion word used to explain a person’s facial expressions may serve as a conceptual framework with which to encode facial features as a concept-congruent representation. Similarly, it has been suggested that these memory biases may be explained by the influence of emotion concepts on the way in which faces are perceived (J. Halberstadt et al., 2009). Such theorizing is consistent with literature on the *categorical perception effect*, in which conceptual knowledge results in the perceptual enhancement of between-category differences and within-category similarities (Harnad, 2003; for a discussion of categorical perception as related to emotion, see Brosch et al., 2010). Future research should examine whether the potential effects of self-generated emotion concepts on memory are mediated by the way emotional stimuli are processed during encoding, by later reconstructive processes, or both.

## Prediction 2: Reducing Conceptual Knowledge Will Reduce the Effects of Emotion on Memory

The theory of constructed emotion predicts that conceptual knowledge is a prerequisite for emotion construction (Barrett, 2014, 2017a, 2017b; Lindquist, 2013; Wilson-Mendenhall et al., 2011). Barrett (2014), for example, stated that the theory of constructed emotion would be falsified by evidence that conceptual knowledge is not required to experience and perceive discrete emotions. In other words, if it could be shown that emotion construction proceeds as expected even in conditions in which conceptual emotion knowledge is inhibited or absent, then the theory of constructed emotion would be falsified. Consistent with the predicted role of conceptual knowledge in emotion construction, ample research demonstrates that facial emotion perception is indeed impaired when availability or access to conceptual emotion knowledge is limited (Bertoux et al., 2020; Calabria et al., 2009; Donges et al., 2014; Gendron et al., 2012; Kamminga et al., 2015; Kumfor & Piguet, 2012; Lane et al., 2000; Lindquist et al., 2006, 2014; Macoir et al., 2019; Nook et al., 2015; Parker et al., 1993; Rosen et al., 2004; Souter et al., 2021).^7^ Relatively less work has experimentally examined effects of concept inaccessibly per se on emotional behavior or experience, but there is evidence that individuals who experience unpleasantness under conditions of increased access to the concept of fear show more risk aversion (a behavior typical of fear) than those individuals who experience unpleasantness under conditions of reduced access to fear (Lindquist & Barrett, 2008a).

### The role of conceptual knowledge in the effects of discrete emotions on memory

Given that the theory of constructed emotion makes a strong prediction that access to conceptual knowledge is necessary for the experience of discrete emotions (Barrett, 2014, 2017a, 2017b; Lindquist, 2013; Wilson-Mendenhall et al., 2011), the CMEM predicts that any effect of discrete emotions on memory (see Fig. 1) should be reduced under conditions in which conceptual knowledge is reduced or absent. Three specific hypotheses follow from this prediction, which we articulate first before describing the methods by which these predictions might be tested. First, it should be the case that in the absence of the availability or accessibility of conceptual knowledge, participants do not exhibit the sort of discrete emotion concept–congruent memory biases described in Prediction 1, as these effects should depend on the categorization of stimuli using discrete emotion concepts. In other words, although the CMEM predicts that attributing a specific emotional concept (e.g., fear) to an event will result in emotion concept–congruent memory biases, these biases should appear only under conditions or in populations in which conceptual knowledge about these emotions is accessible. We acknowledge that participants may still show memory effects of valence and arousal, but if Prediction 1 is correct, emotion-concept knowledge should bias memory beyond valence and arousal.

Second, during memory tests that require participants to make fine-grained mnemonic discriminations between stimuli belonging to different discrete emotion categories (e.g., recognition, source memory, the Mnemonic Similarity Test; Stark et al., 2013), performance should suffer when emotion knowledge is reduced. This prediction follows from the idea that perceiving stimuli as belonging to different conceptual categories results in the perception of greater differences between these stimuli (Brosch et al., 2010; Harnad, 2003). To the extent that the perception of such differences results in stimuli associated with different emotional categories being represented with increased between-stimulus distinctiveness, this ought to benefit memory discrimination. We refer to this phenomenon as *concept-dependent distinctiveness* (see Fig. 1). Consistent with the prediction that the perception of stimuli as being associated with different emotion concepts will increase between-stimulus mnemonic discrimination, a recent study found that providing participants with different emotion labels for faces (“angry” vs “fearful”) does indeed result in faces paired with one label being remembered as more dissimilar than faces with a different label (McMullen et al., 2025). Similarly, in a short-term memory experiment by Fugate et al. (2010), participants who had been taught to identify different primate facial-muscle movements were better able to discriminate between different chimpanzee behaviors, suggesting that acquiring conceptual knowledge about nonhuman facial behaviors results in enhanced short-term memory discrimination for such faces. What remains untested is the CMEM’s novel prediction that the spontaneous process of emotion categorization as mediated by conceptual knowledge will result in the same sort of mnemonic discrimination that has been observed in studies in which emotion concepts are provided by the experimenter.

Last, to the extent that discrete emotions have different effects on memory in certain contexts, reducing emotion knowledge should reduce such differences. For example, discrete emotion effects such as the disgust advantage (Chapman, 2018; Chapman et al., 2013; Ferré et al., 2018; Marchewka, Wypych, Michałowski, et al., 2016; Moeck et al., 2021; Riegel et al., 2022; West & Mulligan, 2021, Experiment 3) should be reduced when access to conceptual emotion knowledge is reduced, because such effects are predicted to be mediated by differences in the features associated with different emotion concepts, which in turn result from concept-dependent emotion construction.

Empirically, predictions regarding the necessity of conceptual knowledge for emotional memory effects may be tested using methods from affective science in which (a) populations deficient in conceptual knowledge about emotions are compared with populations with intact conceptual knowledge or (b) experimental manipulations that modify the availability of conceptual knowledge are applied. With regard to the first approach, in which the emotional memory of populations with deficient conceptual knowledge is examined, one such population is those high in *alexithymia*, a personality trait characteristic of individuals who possess a paucity of conceptual knowledge about discrete emotions (for reviews, see Lindquist & Barrett, 2008b; Taylor & Bagby, 2000). Alexithymia is associated with factors that can impair cognitive development during childhood more generally, such as low socioeconomic status (Karukivi & Saarijärvi, 2014) and deficits in the development of language (Hobson et al., 2018, 2019; K. S. Lee et al., 2025). Thus, the hypotheses discussed regarding alexithymia specifically likely apply to individuals with a relative paucity of conceptual knowledge about emotions due to any cause. Because of their impaired conceptual emotion knowledge, individuals with alexithymia experience difficulties in identifying and describing their emotions (Sifneos, 1973; Taylor & Bagby, 2000). In addition, although such individuals do experience affective sensations (see Lindquist & Barrett, 2008b), they lack the conceptual emotion knowledge necessary for categorization, and as a result experience disruptions in discrete emotion perception (Donges et al., 2014; Lane et al., 2000; Nook et al., 2015; Parker et al., 1993).

A population deficient in conceptual knowledge, broadly speaking, is patients with *semantic dementia* (also known as the temporal lobe variant of frontotemporal dementia), a neurodegenerative disorder characterized by deficits in conceptual knowledge caused by neurodegeneration within the anterior temporal lobes (Hodges & Patterson, 2007). Because conceptual knowledge (including knowledge about emotions) is impaired in patients with semantic dementia, TCE predicts that these individuals’ emotion construction will be impaired as well. Indeed, as with individuals with alexithymia, patients with semantic dementia show marked disruptions in discrete emotion perception (Bertoux et al., 2020; Calabria et al., 2009; Kamminga et al., 2015; Kumfor & Piguet, 2012; Lindquist et al., 2014; Macoir et al., 2019; Rosen et al., 2004).^8^

In addition to testing the above predictions in populations deficient in conceptual knowledge, another way to test whether conceptual knowledge is necessary for the effects of emotion on memory is to manipulate the availability of such knowledge experimentally. For instance, by using a procedure known as *semantic satiation*, it is possible to temporarily inhibit the availability of semantic concepts (for a review, see Black, 2003). Semantic satiation entails having participants repeat a given word many times aloud in a row (e.g., 30 repetitions; Gendron et al., 2012; Lindquist et al., 2006). Eventually, this repetition results in the decoupling of a word’s phonological form from its semantic meaning, rendering the word’s associated concept temporarily inaccessible (Black, 2003). Consistent with the claim that conceptual knowledge is necessary for emotion construction (Barrett, 2014, 2017a, 2017b; Lindquist, 2013; Wilson-Mendenhall et al., 2011), the semantic satiation of emotion words such as “anger” has been shown to disrupt the perception of discrete emotions in facial-muscle movements (Gendron et al., 2012; Lindquist et al., 2006).^9^

Another method of experimentally manipulating the availability of conceptual knowledge is through neurostimulation. Research has shown that by applying repetitive transcranial magnetic stimulation (rTMS) to healthy individuals’ temporal lobes, researchers are able to experimentally induce impairments in conceptual knowledge similar to those seen in patients with semantic dementia (Campanella et al., 2013; Lambon Ralph et al., 2009; Pobric et al., 2007). Similarly, applying transcranial direct current stimulation (tDCS) to the temporal lobes has been shown to affect the availability of conceptual knowledge (for a review, see Joyal & Fecteau, 2016; Pobric et al., 2007). Notably, both temporal rTMS and tDCS have already been shown to affect episodic-memory effects dependent on the availability of conceptual knowledge as discrete emotion effects are predicted to be, with prior research demonstrating that both rTMS and anodal tDCS reduce false memories for semantically related information in the Deese-Roediger-McDermott (DRM) paradigm (Boggio et al., 2009; Díez et al., 2017; Gallate et al., 2009). To our knowledge, past research has not addressed this question with regard to emotional memory, but this would be an interesting avenue of future research.

Having reviewed the predicted effects of conceptual emotion knowledge on the mnemonic effects of discrete emotions, we now demonstrate how these predictions and methods may be combined to generate specific, falsifiable research hypotheses. For instance, by combining the prediction that conceptual emotion knowledge is necessary for concept-congruent memory biases (Prediction 1) with the fact that individuals with alexithymia are thought to be deficient in conceptual emotion knowledge, the CMEM generates the hypothesis that individuals high in alexithymia will show reduced concept-congruent memory biases relative to those low in alexithymia. Similarly, because discrete emotion effects (such as the disgust advantage) are predicted to depend on the availability of discrete emotion concepts such as disgust, and because the availability of such semantic concepts may be inhibited through temporal neurostimulation, the CMEM generates the hypothesis that the disgust advantage should be reduced under conditions of inhibitory temporal neurostimulation, compared with sham stimulation. Although by no means an exhaustive combination of the predictions and methods listed above, these hypotheses illustrate the value of adopting a constructionist perspective when considering the conditions under which discrete emotions may or may not affect later memory.

### The role of conceptual knowledge in the effects of valence and arousal on memory

Whereas constructionist theories make strong predictions about the role of conceptual emotion knowledge in discrete emotion construction, the role of conceptual knowledge in the perception of affect (i.e., valence and arousal) is an ongoing question. First, it should be emphasized that conceptual knowledge is not thought to be necessary for the perception of affect in an absolute sense, as evidenced by the fact that one may come to view stimuli as simply being positive or negative because of relatively automatic processes that do not require the experience of categorical emotion states, such as classical conditioning (Lindquist, 2013). The question, however, is whether the construction of discrete emotion states might influence the specificity or intensity with which one experiences sensations of valence and arousal during an emotional episode.

Articulation of constructionist theory provides some suggestion that categorization may fundamentally transform the meaning of affective sensations. Barrett (2005), for example, wrote that by parsing affective sensations into discrete emotional experiences through the process of emotion construction, affect is imbued with “intention or emotional aboutness” (p. 270). Such intention and aboutness may mean that the affective features of valence and arousal are represented in a more specific, stimulus-directed manner when the perceiver has access to conceptual knowledge. Consistent with this assertation, some evidence has suggested that in addition to experiencing disruptions in discrete emotion perception, both individuals with alexithymia (De Gucht & Heiser, 2003; Grabe et al., 2004) and semantic dementia (Mendez, 2021) tend to attribute affective sensations to physiological causes (i.e., somatization) rather than to external stimuli. This raises the possibility that sensations of valence and arousal may be experienced as less stimulus-specific and more generalized by populations with deficits in conceptual knowledge. Additional empirical support for the notion that conceptual knowledge may interact with the representation of affect is seen in studies demonstrating divergent effects of affect on behavior (Lindquist & Barrett, 2008a; Lee et al., 2018) and experience (Lee et al., 2018) depending on the presence of accessible concept knowledge of “fear.” Additionally, to the extent that qualities such as negative valence and high arousal are associated with one’s representation of specific emotion concepts such as fear, it may be predicted that the perception of a stimulus as being frightening will result in the stimulus being perceived as more negative and arousing than it would have been in the absence of access to conceptual knowledge of “fear.” Suggestive of this hypothesis is evidence that people primed with the concept “fear” (vs. a neutral control) show a greater startle reaction to aversive images (Oosterwijk et al., 2010). Furthermore, people who are higher in *emotional granularity*—who differentiate more among their emotions in daily life and who are thought to experience their emotions more discretely through greater access to concept knowledge (Hoemann, Nielson, et al., 2021; Lindquist & Barrett, 2008b)—show even greater sympathetic arousal during a stressor than individuals who are lower in differentiation (Bonar et al., 2023). This might tend to support the possibility that discrete emotional construction may amplify the experience of affect.

If access to conceptual knowledge renders affective sensations that are more specific and intense, reductions in conceptual knowledge should reduce what we refer to here as the “typical effects of emotion on memory,” which have historically been attributed to valence and arousal (i.e., emotionally enhanced memory, emotionally enhanced vividness, memory trade-off effects). Given the state of the current literature on this topic and the logic outlined above, we believe the assumption underlying this specific prediction to be sensible at present. At the same time, it should be emphasized that this assumption is modular with respect to the validity of the CMEM, broadly speaking. Should future research in affective science indicate that the availability of conceptual knowledge does not affect the specificity or intensity with which one perceives valence and arousal, the rest of CMEM’s predictions will be unaffected.

Thus, the CMEM predicts that the typical effects of emotion on memory should be weaker for individuals with reduced conceptual emotion knowledge. Indeed, ample evidence already exists to support this prediction. With regard to semantic dementia, evidence suggests that—in contrast to healthy older adults and patients with Alzheimer’s disease—patients with semantic dementia do not show emotionally enhanced memory (Kumfor et al., 2013) and are less likely than controls to recall emotional details when retrieving recent autobiographical memories (Irish et al., 2011). With regard to alexithymia, ample evidence suggests that individuals with alexithymia show reduced memory for emotional but not neutral information (for a review, see Apgáua & Jaeger, 2019). For example, Meltzer and Nielson (2010) found that participants high in alexithymia recalled fewer negative words than participants low in alexithymia. Similarly, Ridout et al. (2020) found evidence that alexithymia is associated with reduced memory of emotional facial expressions and emotionally charged social interactions (see also Donges & Suslow, 2015). Such results suggest that, as predicted by the CMEM, the beneficial effects of emotion on memory are selectively reduced for individuals with alexithymia.

To date there does not appear to be a single commonly accepted explanation for reduced emotional memory in individuals with alexithymia. Some have argued that these deficits result from the fact that these individuals perceive emotional information as less salient because of their less well-integrated emotion schemas (Luminet et al., 2006; Ridout et al., 2020; Suslow et al., 2003); in contrast, others have explained these effects as being due to deficits in cognitive control (Dressaire et al., 2015), or, in the case of memory for emotional facial expressions, as due to the challenges that individuals with alexithymia experience in communicating their own feelings (Donges & Suslow, 2015). The CMEM offers a unifying explanation that is largely consistent with the impoverished-emotion schema-based explanation of these findings (but see footnote 6). That is, the CMEM predicts that alexithymia selectively impairs memory for emotional information because deficits in conceptual emotion knowledge disrupt the attribution of meaning to affective stimuli that would normally occur during emotion construction (Barrett, 2006, 2017a; Barrett et al., 2015). Thus, although individuals with alexithymia may have a preserved experience of affect (Lindquist & Barrett, 2008b), the experience of affective sensations alone may be insufficient to allow one to perceive external stimuli as emotional in a meaningful sense. Such stimuli may therefore not be processed in ways which would normally result in emotionally enhanced memory (see Fig. 1). Empirically, the validity of this explanation could be assessed by testing whether the reduced emotional memory of individuals with alexithymia is mediated by reductions in their personal ratings for normatively emotional stimuli of valence, arousal, and specificity or distinctiveness.

An alternative—or perhaps complementary—explanation of such effects is that linking stimuli to a specific discrete emotion concept, as mediated by conceptual knowledge, results in memory enhancements beyond what one would observe if the same stimulus had been perceived as simply being valenced and arousing. As discussed above, the attribution of emotional meaning to stimuli might be expected to enhance their degree of self-relevance (Barrett, 2017a; Feldman et al., 2024; Shaffer et al., 2022), which, if true, ought to result in better memory (Czienskowski, 1997; Symons & Johnson, 1997). Additionally, the perception of a stimulus as being associated with a specific discrete emotion may result in memory enhancements by increasing the distinctiveness with which said stimulus is represented (see also Fig. 1). Broadly speaking, such possibilities illustrate that, at present, it is unclear how much of the typical effects of emotion on memory are due to valence and arousal per se as opposed to some combination of affect and discrete emotion. It is our hope that the current article provides a framework for future investigations to tease apart these issues.

With regard to experimental methods of modifying the availability of conceptual knowledge, the CMEM predicts that such methods should constrain the effects of emotion on memory. Because no study to our knowledge has investigated whether methods such as semantic satiation of emotion concepts or neurostimulation of brain regions implicated in conceptual knowledge impacts the typical effects of emotion on memory, this novel prediction remains to be tested. However, Gendron et al. (2012) used a repetition-priming paradigm in which they showed that semantic satiation of the word “anger” prior to viewing a posed, scowling face prevented that face from priming itself moments later. That is, the inhibition of conceptual knowledge presumably influenced perceptual encoding of the first instance of the face, which impaired short-term visual memory. Future research should extend these effects to paradigms in which long-term encoding and retrieval could be examined.

Although some prior research suggests that populations with reduced conceptual knowledge show reductions in emotionally enhanced memory, it remains to be seen whether the participant-level characteristics and experimental manipulations discussed here also reduce the vividness (Bowen et al., 2018; Buchanan, 2007; Kensinger & Ford, 2020; Kensinger & Schacter, 2016; Levine & Pizarro, 2004; Ochsner, 2000; Phelps & Sharot, 2008; Talmi, 2013) and memory trade-off effects (Dolcos et al., 2017; Kensinger, 2009a, 2009b; Kensinger et al., 2007; Levine & Edelstein, 2009; Levine & Pizarro, 2004; Mather & Sutherland, 2011) associated with emotion. Although preliminary evidence indicates that individuals with alexithymia remember emotional information with a reduced degree of recollection (Luminet et al., 2006; Vermeulen & Luminet, 2009), it is currently unknown whether semantic dementia/aphasia, semantic satiation, or neurostimulation of the temporal lobes have similar effects on the phenomenology with which emotional events are remembered. Furthermore, to our knowledge no study has examined whether the memory trade-off effect is dependent on conceptual knowledge as predicted by the CMEM using any of these methods. Broadly speaking, the utility of the CMEM with respect to the mnemonic effects of valence and arousal can be seen as specifying for whom and under which circumstances the typical effects of emotion on memory may or may not manifest.

## Prediction 3: Variability in Emotion Construction Will Affect Emotional Memory

Theories of emotion can be distinguished in terms of how they explain variability between instances of a specific emotion. On the one hand, accounts such as basic-emotion theory treat variability as epiphenomenal and due to extraneous factors such as emotion regulation, cultural norms about which emotions should be expressed or emphasized (but not necessarily experienced), or differences in intensity between instances of the same emotion (e.g., Ekman et al., 1969; Tracy, 2014). Constructionist theories, on the other hand, emphasize the importance of variability as fundamental to the very nature of emotion (Barrett, 2014). Indeed, the theory of constructed emotion assumes variability in the manifestations of emotion both within and between people and within and between different instances of emotion categories “to be the norm, rather than a nuisance to be explained after the fact” (Barrett, 2017b, p. 16). Growing evidence backs up these hypotheses by documenting extreme between- and within-individual variability across instances of emotion (e.g., Hoemann et al., 2020; McVeigh et al., 2024). Accordingly, this theory conceives of different instances of a given discrete emotion as belonging to a population of variable category instances that differ from one another in important, context-specific ways (Barrett, 2015, 2017a).

Constructionist theories predict substantial variability in how emotion construction will proceed, because emotion construction is believed to be situated within the context of the perceiver’s current circumstances, prior experiences, and culture (Barrett, 2014; Barrett et al., 2011). Because such variation is predicted to affect how affective sensations and emotion concepts are attributed to stimuli during emotion construction, the CMEM predicts that this variability will also affect how these stimuli are remembered. Here we focus on three sources of variability that are predicted to serve as ongoing contexts for emotion construction: intraindividual variability between situations, interindividual variability between people, and variability between cultures.

### Situational variability in emotion construction

The theory of constructed emotion predicts that a stimulus’s affective and emotional value will depend on situational factors, so that a stimulus might be perceived as more or less arousing, positive, negative, or evocative of a certain emotion depending on the situation within which it is perceived (Barrett, 2006). In line with this prediction, there exists an extensive body of evidence demonstrating that context has a strong influence on the emotions perceived in facial-muscle movements (Aviezer et al., 2008, 2009, 2011, 2017; Ensenberg-Diamant et al., 2025; Goel et al., 2024; Hassin et al., 2013; Israelashvili, Hassin, & Aviezer, 2019). Also, consistent with the prediction that situational variability influences emotion construction, research has shown that participants’ affective and physiological responses to emotional stimuli depend on the situational context within which they are framed (Fernandes et al., 2019; Foti & Hajcak, 2008; Kirk et al., 2020; Santos et al., 2023). For example, Fernandes et al. (2019) found that participants rated images of hands covered in ambiguous substances as more negatively valenced, arousing, and disgusting if these images were framed as being photos of someone suffering from a gastrointestinal disease. Given that contextual framing manipulations have been shown to impact emotion perception and affect perception, it may be predicted that such factors will also affect memory. For instance, because contextual-framing manipulations imbue stimuli with affective and emotional meaning, it may be predicted that nonemotional stimuli will exhibit emotional-memory effects if framed in a way that makes them emotional within a given situational context. Consistent with this prediction, nonemotional stimuli that are framed as having been touched by contaminated hands are better remembered than those framed as being touched by noncontaminated hands (Bonin et al., 2019; Fernandes et al., 2017, 2021). Conversely, it may also be possible to reduce or eliminate the emotional-memory effects associated with normatively emotional stimuli by framing them in contexts that reduces their emotionality (e.g., by presenting an image of a car accident as an artificial recreation for a movie rather than as an actual car crash).

In addition to demonstrating that situational context can imbue stimuli with affect and emotion, research drawing on constructionist theories has also demonstrated that situational features explain heterogeneity that exists between different instances of the same emotion. For example, Wilson-Mendenhall et al. (2011) reported that functional magnetic resonance imaging (fMRI) data demonstrate that the brain regions associated with fear and anger differ within these emotions depending on whether fear and anger were experienced in situations associated with physical danger or with social evaluation. Likewise, Kveraga et al. (2015) found that the threat appraisals and fMRI activity associated with fear differed depending on whether participants were responding to direct threats, indirect threats, or past threats (see also Wang et al., 2024). Last, McVeigh et al. (2024) found that the relationship between patterns of physiological activity and levels of fear evoked by videos of spiders, heights, and social evaluation were best accounted for by a model that took into account differences between the three situation types as well as interindividual variability.

Taken together, these results suggest that important differences exist between instances of the same emotion depending on the situational context within which they are experienced. Given these results, it may be predicted that, rather than specific emotions such as fear or disgust exhibiting one-to-one effects on memory, the mnemonic effects of a given emotion will depend critically on the situation within which it is experienced. For example, given research demonstrating that instances of fear differ depending on whether the perceiver is experiencing fear of physical danger or fear of social evaluation (Wilson-Mendenhall et al., 2011), one might predict that the specific contextual features encoded into memory will differ in these situations, with sources of physical danger (e.g., weapons, predators) being better remembered for the former and the reactions and behaviors of others being better remembered for the latter. Broadly speaking, the general prediction that the effects of emotion on memory depend on situational context dovetails with prior research demonstrating that situational context is selectively encoded into memory during emotion perception (Barrett & Kensinger, 2010), highlighting the possibility that contextual features and emotion may interact in important ways with respect to episodic memory.

### Interindividual variability in emotion construction

Constructionist theories predict that individuals differ considerably in how they construct their emotions. As briefly discussed above, one construct that has received considerable attention as a source of interindividual variability in emotion is emotional granularity,^10^ or the extent to which an individual represents emotions in a situation-specific and differentiated manner (Barrett & Bliss-Moreau, 2009; Hoemann, Nielson, et al., 2021; Lindquist & Barrett, 2008b; Tugade et al., 2004). A common approach to measuring granularity is to ask participants to rate episodes from their daily lives in terms of various emotional terms (e.g., anger, fear, sadness) across multiple events. Higher intercorrelation between ratings of different same-valenced emotions within episodes indicates lower granularity insofar as this indicates that individuals do not differentiate among same-valence emotion categories when rating events during their daily lives. For example, whereas a highly granular individual might feel angry after failing to receive a promotion, afraid after getting bad news about health, and sad after receiving negative feedback, a less granular individual may not make such fine-grained distinctions and may instead report similar levels of each emotion within these events. The concept of emotion granularity highlights the fact that people may not experience the same emotions in reaction to the same stimuli. Rather, constructionist theories predict that individual differences in constructs such as emotional granularity result in meaningful variation in emotion construction between individuals (Barrett, 2017a; Hoemann, Nielson, et al., 2021; Lindquist & Barrett, 2008b). Differences in emotional granularity are thought to arise from variation in the structure of individuals’ conceptual representations of emotions. More specifically, it is theorized that highly granular individuals either possess more differentiated emotion concepts or are more likely to use those concepts in the moment, allowing these individuals to represent their emotions in a nuanced, situated manner (Hoemann, Nielson, et al., 2021; Lindquist & Barrett, 2008b).

Research suggests that the effects of emotional granularity go beyond verbal reports of emotional experiences and extend to the level of differentiation with which emotional stimuli and events are processed and perceived. For example, Israelashvili, Oosterwijk, et al. (2019) found that individual differences in negative emotional granularity (i.e., the extent to which one’s experiences of different negative emotions are differentiated) were associated with higher performance on a task of facial emotion recognition. Additionally, an electroencephalography (EEG) study in which participants viewed emotional images found that granularity was related to differences in alpha and gamma synchrony (associated with access to conceptual knowledge and affective processing, respectively; Benedek et al., 2011; Güntekin & Başar, 2014; Jaušovec & Jaušovec, 2005), and that highly granular participants exhibited increased N2 amplitudes (associated with executive control; Folstein & Van Petten, 2008) and late positive potential amplitudes (associated with motivated attention; Hajcak et al., 2009) compared with less granular participants (J. Y. Lee et al., 2017). These findings demonstrate that individuals who differ in granularity process the same emotional stimuli differently, and they were interpreted by J. Y. Lee et al. (2017) as evidence that highly granular individuals process emotional information in a more complex and differentiated manner. Similar associations between granularity and differentiation in emotion representation have been demonstrated in daily life, with highly granular individuals demonstrating higher experiential diversity (Hoemann, Lee, et al., 2023) and higher context specificity in emotion-related cardiorespiratory physiology (Hoemann, Khan, et al., 2021) than individuals low in granularity. Taken together, such research suggests that highly granular individuals tend to represent emotional stimuli and events with greater differentiation and context specificity than those low in granularity.

If highly granular individuals represent emotional stimuli in a more differentiated (i.e., distinctive) manner (J. Y. Lee et al., 2017; Lindquist & Barrett, 2008b; Wang et al., 2020), such individuals should show larger benefits of emotion on memory. This prediction is motivated by classic research demonstrating that stimuli that are processed in a more distinctive manner are ultimately better remembered, as such processing results in the formation of memory traces that are more discriminable (Hunt & McDaniel, 1993; Jacoby & Craik, 1979). Additionally, because emotionally enhanced memory is thought to be partially mediated by distinctiveness (Talmi, 2013; Talmi & McGarry, 2012; Talmi et al., 2007), any variable that increases the distinctiveness of emotional stimuli ought to increase their mnemonic advantage.

The prediction that highly granular individuals will show increased emotional memory was investigated in the first author’s doctoral dissertation (West, 2023). In this study, participants completed multiple end-of-day reports of their emotional experiences during episodes from their daily lives as well as laboratory tests of episodic memory for emotional images and DRM word lists.^11^ Results showed that participants high in granularity for negative emotions in daily life exhibited stronger valence-based memory effects for emotional images than those low in negative granularity. Such results suggest that the effects of emotion on memory depend on one’s level of granularity, at least for certain visual stimuli. In light of these preliminary results, researchers are encouraged to conduct additional investigations on the mnemonic effects of granularity on emotional information. In particular, because granular individuals have been conceived of as experts in representing complex and nuanced emotions (Hoemann, Nielson, et al., 2021), future research should investigate the possibility that granularity exhibits stronger effects on memory for information that requires more nuanced emotion construction, such as emotionally ambiguous materials (Brainerd, 2018; Brainerd et al., 2021) or emotionally complex autobiographical memories.

Although we have focused mostly on how emotional granularity might affect memory at the behavioral level, it is also possible that differences in granularity relate to the neural processes mediating emotional memory as well. As already discussed, prior EEG research has shown that individual differences in granularity are related to electrocortical measures such as event-related potentials and event-related synchrony when processing emotional images (J. Y. Lee et al., 2017; Wang et al., 2020). Because such research suggests that differences in granularity are associated with variability in the neural representation of emotional information, it is possible that granularity is likewise associated with variability in the neural correlates of emotional memory. For instance, given that granularity is thought to allow for more distinctive representation of emotional information, it might be predicted that item-specific patterns of neural activation within brain regions involved in affect and emotion representation will be more differentiated from one another (i.e., less interchangeable) for highly granular individuals, and that this increased neural distinctiveness will predict enhancements in emotional memory. This prediction could be evaluated using multivariate analyses of fMRI data such as representational similarity analysis (Dimsdale-Zucker & Ranganath, 2018; Kriegeskorte et al., 2008; Sommer & Sander, 2022), and if true, would suggest that highly granular individuals’ increased distinctiveness in their neural representation of emotion is associated with enhanced memory for emotional information.

Individual-level characteristics other than emotional granularity likely also impact emotional memory through their effects on emotion construction. An essential prediction of the theory of constructed emotion is that, because a stimulus’s physiological consequences are predicted on the basis of one’s learning history, the computation of valence, arousal, and emotion will depend on the perceiver’s prior experiences (Barrett et al., 2015; Lindquist, 2013). By highlighting the importance of idiosyncratic variability in emotion and affect perception, constructionist theories underscore the need to take seriously the possibility that individuals may differ considerably in their perception of identical emotional stimuli. Indeed, recently Westlin et al. (2026) demonstrated that emotion labels based on normative ratings of images and film clips do a poor job of describing the emotional experiences of individual participants, providing strong evidence against the basic-emotion view that affective stimuli reliably and exclusively evoke specific discrete emotions across individuals.

To the extent that such variability affects cognitive processes, such as memory, it may be warranted to adopt an idiosyncratic approach to operationalizing affect and emotion—one in which experimenters rely not on normative ratings to define the properties of their stimuli but instead on the idiosyncratic, person-specific perceptions of the individuals within a given study. Having observed substantial idiosyncratic variability in discrete emotion perception (see also Ensenberg-Diamant et al., 2025), Westlin et al. (2026) likewise advocated for “idiographic analytical approaches that explicitly model subject-level variability” (p. 13). Unpublished work in our lab suggests that this approach may indeed be fruitful, as an image-recall experiment revealed that participants’ own idiosyncratic ratings of valence were substantially better at predicting their subsequent recall performance than normative ratings of the same images (West, 2023).^12^ Ongoing work in our lab is investigating this issue.

Central to the CMEM is the prediction that the factors that impact emotion construction will impact emotional memory. Thus, although cognitive factors such as distinctiveness and relatedness are typically conceived of as inherent properties of emotional stimuli (Talmi, 2013; Talmi & Moscovitch, 2004; Talmi et al., 2007), the CMEM raises the possibility that such properties might be better thought of as emergent properties arising during emotion construction and that factors such as emotional granularity may place important perceiver-dependent constraints on such properties. The hypotheses regarding individual differences in emotion construction that follow from the theory of constructed emotion and from the CMEM may prove particularly valuable in advancing memory research, given that the majority of emotional-memory research to date has investigated how emotional stimuli are remembered on average, without considering the ways that individuals differ in their emotion perception (but for exceptions, see Dolcos et al., 2017, 2020; Hamann & Canli, 2004; Waring et al., 2009).

There are no doubt additional sources of variability in emotion construction that should be investigated in future research. After all, variability in emotion is not restricted to the level of the individual or the current situation. In the following section, we consider the mnemonic effects of emotional variability at another level of analysis: that of culture.

### Cultural variability in emotion construction

Because constructionist theories propose that emotion construction depends on learned emotion concepts, these theories predict that differences in the nature of the emotion concepts transmitted to individuals by their culture should affect emotion construction. In other words, like situational factors and individual differences in conceptual knowledge, culture provides an ongoing context within which emotion construction is situated (Gendron et al., 2018). Thus, unlike basic-emotion theories that predict that emotional experiences and expressions are universal (Ekman, 1999; Ekman & Cordaro, 2011; Tracy, 2014), constructionist theories predict that the emotions one experiences and perceives in others depend on social concepts that are culturally relative (Barrett, 2014, 2017a). Consistent with this claim, research has shown that when using unconstrained methods, the emotions individuals perceive in facial-muscle movements show substantial cross-cultural variability (for reviews, see Gendron, 2017; Gendron et al., 2018). In this section, we consider the mnemonic implications of such variability.

A review of the extensive literature on cross-cultural differences in emotion is beyond the scope of the current article. Instead, we focus on one way that cultures differ with respect to emotions to demonstrate the utility of considering cross-cultural differences in emotional-memory research. In particular, we consider cross-cultural differences in the extent to which emotional experiences and perceptions are influenced by ongoing social context. Because most of the cross-cultural emotion research on this topic has focused on the perception of facial expressions, such research is the focus of this section. As with any research described here related to facial emotion perception (e.g., research demonstrating that emotion concepts bias memory for facial expressions, Doyle & Lindquist, 2018, Experiments 1 and 2; Fugate et al., 2018; J. Halberstadt, 2005; J. B. Halberstadt & Niedenthal, 2001; J. Halberstadt et al., 2009), we predict that the same mechanisms at play during emotion construction that lead to cross-cultural differences in facial emotion perception will also impact emotional reactions and memories for nonfacial stimuli (see footnote 7).

Cross-cultural research has suggested that Westerners tend to view emotions as phenomena that occur within individuals, whereas Easterners tend to view emotions as phenomena that occur between people within the context of social interactions (for a review, see Mesquita, De Leersnyder, & Boiger, 2016). In order to investigate the influence of social context on cross-cultural differences in emotion perception, Uchida et al. (2009) conducted a series of studies in which American and Japanese participants made judgments about Olympic athletes. In support of the claim that Easterners are more likely to think of emotion as something that occurs between people, the authors found that Japanese participants inferred more emotion in athletes who were shown with others, whereas Americans inferred more emotion in athletes who were pictured alone. This suggests that the degree to which an event is perceived as emotional depends on an interaction between one’s culture and the social context of the event. Also consistent with the idea that cultures differ in the extent to which social context influences emotion construction, research has demonstrated differences between cultures in the extent to which emotion perception is affected by the emotions of task-irrelevant others. More specifically, research has shown that whereas Easterners’ emotion ratings of a target face are influenced by the expressions of peripheral, task-irrelevant people, Westerners’ ratings are not (Masuda et al., 2008, 2012).

Taken together, prior research has demonstrated that cultures differ in the extent to which emotion construction is influenced by the ongoing social context of an event. If one takes the constructionist approach of conceiving of emotions as emergent phenomena constructed using culturally relative emotion concepts (Barrett et al., 2015; Wilson-Mendenhall et al., 2011), whether one conceptualizes emotions as social or asocial phenomena might be thought of as an aspect of conceptual emotion knowledge that differs between cultures. Because the CMEM predicts that variability in the conceptual representation of emotions should affect emotional memory, this model predicts that such cross-cultural differences will impact the way that members of different cultures remember emotional information and events.

Two predictions regarding emotional memory can be made from the premise that cultures differ in the extent to which emotion construction is influenced by social context. First, if cultures vary in the extent to which the presence of others influences the degree to which an event is perceived as emotional (Levenson et al., 1992; Uchida et al., 2009), then the presence of others will affect participants’ emotional memory differently, depending on their culture. For example, compared with American participants, Japanese participants may exhibit stronger emotional memory effects for social compared with asocial stimuli. Second, if Easterners’ emotion construction is more influenced by social context than Westerners’ (Masuda et al., 2008, 2012), then details of the social context of an emotional event will have a stronger influence on Easterners’ memories than Westerners’. In particular, this view predicts that stimuli surrounded by people reacting in emotional ways will show stronger emotional memory effects for Easterners compared with Westerners. Similarly, it is predicted that Easterners will exhibit stronger concept-congruent memory biases for stimuli surrounded by people displaying stereotypical expressions of a specific emotion (e.g., fear) than will Westerners (see Prediction 1).

Although far from exhaustive, the predictions discussed in this section regarding the potential influence of culture on emotional memory illustrate the utility of considering the mnemonic consequences of cross-cultural differences in emotion construction. In addition to testing such predictions, future work should consider as well the role of other well-established cross-cultural differences in emotion. For example, research has shown that culturally reinforced emotions tend to be experienced with greater prevalence and intensity (Boiger & Mesquita, 2012; Mesquita, Boiger, & De Leersnyder, 2016; Mesquita, De Leersnyder, & Boiger, 2016). Thus, it may be that the effects of a given emotion on memory will be stronger in cultures where that emotion is more salient compared with cultures where it is less salient. For instance, because the experience of anger is thought to be up-regulated in the United States, whereas shame is thought to be up-regulated in Japan (Boiger & Mesquita, 2012; see also Kirmayer, 1991; Kleinknecht et al., 1997; Tanaka-Matsumi, 1979), it can be predicted that anger will have a stronger effect on the memories of American individuals, whereas shame will have a stronger effect on the memories of Japanese individuals.

Although it is generally the case that emotional information is seen as more conceptually interrelated than neutral information (Talmi & Moscovitch, 2004; Talmi et al., 2007), the extent to which this is true may depend on whether or not emotional stimuli are thought of as exemplars of a superordinate and distinct emotion category. Because research suggests that certain cultures may not possess an explicit concept of emotion in their lexicon (e.g., Samoans, Tahitians, Gidjingali aborigines; see Russell, 1991), it may be that such cultures do not perceive emotional stimuli as being conceptually interrelated in the same way that Western cultures do.^13^ If so, this would have important implications for emotional-memory research, given that increased relatedness is thought to partially explain the emotionally enhanced memory effect (Talmi, 2013). In particular, the lack of a distinct concept for emotions among Samoans suggests that they may not represent emotional stimuli as being conceptually related (Russell, 1991) and may not show the corresponding mnemonic effects of relatedness for emotional stimuli (Russell, 1991; Talmi, 2013; Talmi & Moscovitch, 2004; Talmi et al., 2007).

As illustrated by the fact that cultures differ in whether they have a distinct word for “emotions” (Russell, 1991), cross-cultural differences in emotion lexicons provide important information regarding differences in the nature of emotion concepts between cultures. In this way, emotion lexicons may be thought of as indicators of the collective emotion knowledge propagated through a cultural context. Consequently, an examination of the emotion words present in a culture’s lexicon will likely prove fruitful when generating hypotheses regarding cross-cultural differences in emotional memory.

As an example, because the CMEM predicts that the mnemonic effects of discrete emotions depend on the nature of the discrete emotion concepts accessed during emotion construction, differences between cultures in these emotion concepts as reflected by their emotion lexicons may result in differences in memory for stimuli associated with those emotions. As a specific example, the absence of a translationally equivalent word for the English “disgust” in Polish (Ba˛k, 2023; Russell, 1991; Wierzbicka, 1986) suggests that this specific emotional state (as conceptualized by English speakers) may be less salient in Polish culture. If so, this raises the intriguing possibility that although stimuli conceptualized as disgusting by Americans (Chapman, 2018; West & Mulligan, 2021, Experiment 3), Canadians (Chapman et al., 2013), Australians (Moeck et al., 2021), and Spaniards (Ferré et al., 2018), are more memorable than those conceptualized as frightening (i.e., the disgust advantage), this difference in memorability may be reduced for Polish participants. Although one research group has demonstrated some evidence that disgusting stimuli are better remembered than frightening stimuli by Polish participants (Marchewka, Wypych, Michałowski, et al., 2016; Riegel et al., 2022; but see Marchewka, Wypych, Moslehi, et al., 2016), it is an open question for future cross-cultural research whether such effects are equal to or smaller than those observed in other cultures when using the same procedures and materials. This prediction raises the broader possibility that the effects of specific discrete emotions on memory are not universal, but rather situated within cultural contexts that differ in how emotions are learned, reinforced, and enacted.

The prediction that cross-cultural differences in emotion construction will result in differences in emotional memory has important implications for the generalizability of emotional-memory research. Central to constructionist theory is the idea that cross-cultural variability is a meaningful and fundamental aspect of emotion (Barrett, 2017a; Boiger & Mesquita, 2012; Gendron, 2017; Gendron et al., 2018; Hoemann, Gendron, et al., 2023; Mesquita, 2022; Mesquita & Boiger, 2014; Mesquita, Boiger, & De Leersnyder, 2016; Mesquita, De Leersnyder, & Boiger, 2016). Likewise, the CMEM predicts that the emotion concepts that enable emotion construction are culturally situated and that cross-cultural differences in emotion concepts will affect how emotional stimuli are remembered. Care must be taken when attempting to generalize emotional-memory findings beyond the Western samples that dominate the extant research. Indeed, recent research has begun to investigate the ways in which culture places boundary conditions on emotional memory effects. For example, although American participants show impaired memory for neutral backgrounds previously paired with emotional images, this memory trade-off effect appears to be absent for Turkish participants (Gutchess et al., 2018). These findings provide support for the idea that certain memory effects that are often thought of as general facts about emotional memory may in fact apply only to some cultures but not to others. Such findings caution against the assumption that findings regarding emotional memory are universal, and they reinforce the need to determine whether the effects and theories that describe Westerners’ emotional memories apply to non-Western cultures as well.

## A Constructionist Interpretation of Existing Discrete Emotion Effects on Memory

Recently, memory researchers have become increasingly interested in the possible effects of discrete emotions on memory, with such investigations drawing on basic-emotion theories (Ekman, 1999; Ekman & Cordaro, 2011; Tracy, 2014) and causal-appraisal theories (see Moors, 2014; Roseman & Smith, 2001). Likewise, many of the CMEM’s novel predictions have to do with the effects that categorizing affective sensations into discrete emotional states will have on memory (e.g., conceptual-congruent memory, concept-dependent distinctiveness). Crucially, however, although our model predicts that accessing discrete emotion concepts during emotion construction will influence how participants remember emotional information, the CMEM predicts that such effects will be *situated*, by which we mean that discrete emotions will not have specific and consistent (i.e., one-to-one) effects on memory or its neural and physiological correlates, but will instead have contextually sensitive effects that vary within individuals depending on situational factors, between individuals depending on their conceptual knowledge and prior experiences, and between cultures depending on cross-cultural differences in emotion concepts (Barrett, 2009).

Emotional-memory research inspired by basic-emotion and causal-appraisal theories is undoubtably valuable in that it has encouraged memory researchers to expand upon the ways that emotion is operationalized, conceptualized, and investigated. However, the conclusions reached when adopting these perspectives stand in stark contrast to the assumptions of the CMEM. In particular, our model predicts that the effects of discrete emotion on memory are contextually situated and variable, but perspectives such as basic-emotion theory predict that such effects are context-free and universal.

The theory of constructed emotion emphasizes that when we researchers demonstrate an effect of a specific emotion on cognition, behavior, or physiology, we should not assume that this result reflects the specific effect that this emotion will have during every instance of that emotion. Indeed, because the theory of constructed emotion acknowledges that emotional experiences have both dimensional and categorical components, finding differences between emotions within a specific experimental context does not falsify this theory or provide exclusive support for competing emotion theories (Barrett, 2015). Instead, the CMEM provides an alternative explanation for discrete emotion effects: that such effects are the result of differences in the patterns of information processing associated with contextually sensitive instances of discrete emotions, which are themselves drawn from variable populations of possible category instances. Similarly, rather than interpreting a particular pattern of neural activation as the specific way in which the brain will represent every episode associated with a particular emotion in memory, such patterns may instead be viewed as summaries of the contextually sensitive processes employed by participants when constructing a particular instance of emotion, given the constraints of the current experimental task (Wilson-Mendenhall et al., 2011, 2013).

Notably, the CMEM diverges from alternative accounts of discrete emotion effects by predicting that the existence of discrete emotion effects will depend critically on contextual factors, such as features of the current situation, the availability of conceptual knowledge, and differences in the nature of conceptual knowledge about emotions between individuals and cultures (Barrett, 2009, 2015; Barrett et al., 2015). In particular, and as discussed above, the CMEM predicts that discrete emotion effects, such as the disgust advantage, will be stronger when conceptual knowledge regarding discrete emotions is accessible (e.g., for individuals low in alexithymia; Lindquist & Barrett, 2008b; Taylor & Bagby, 2000; see Prediction 1), and in contexts where a given discrete emotion is more salient (e.g., in cultural contexts where a given emotion is up-regulated; Boiger & Mesquita, 2012; Mesquita, Boiger, & De Leersnyder, 2016; Mesquita, De Leersnyder, & Boiger, 2016). In this way, constructionist interpretations of discrete emotion effects may inspire fruitful investigations aimed at identifying the contextual factors associated with variability in how discrete emotions affect later memory. Testing such predictions will inform not just research on emotional memory, but also research on the nature of emotions more generally.

## Conclusion

For more than a century, researchers have debated the nature of emotion (Gendron & Barrett, 2009). As a result, competing theories have developed within affective science, along with an extensive literature testing the validity of these theories. To the extent that such theories represent mutually exclusive explanations of the very nature of emotion, the theoretical perspective one adopts when conducting emotion research will no doubt influence the research questions that are asked, the studies that are conducted, and how the results of such studies are interpreted. To date, much memory research has adopted assumptions from affective science based on the documented dimensional features underlying emotion (valence and arousal; Russell, 1980; see Kensinger & Corkin, 2004) or by applying the perspectives of basic-emotion theories (Ekman, 1999; Ekman & Cordaro, 2011; Tracy, 2014) and causal-appraisal theories (see Moors, 2014; Roseman & Smith, 2001) to emotional memory (e.g., Chapman, 2018; Chapman et al., 2013; Ferré et al., 2018; Kaplan et al., 2012, 2016; Levine & Burgess, 1997; Levine & Edelstein, 2009; Levine & Pizarro, 2004; Marchewka, Wypych, Michałowski, et al., 2016; Riegel et al., 2022; West & Mulligan, 2021, Experiment 3).

The goal of the current article is to demonstrate the utility of constructionism as a novel paradigm for conducting emotional-memory research (Barrett, 2014, 2017a, 2017b; Barrett et al., 2025; Cunningham et al., 2013; Lindquist, 2013; Satpute et al., 2020). In particular, the CMEM draws upon Barrett and colleagues’ theory of constructed emotion, which posits that emotions are emergent mental events created during emotion construction, during which perceivers use conceptual knowledge to make meaning of external sensory information and internal affective sensations in context (Barrett, 2017a, 2017b). With regard to existing empirical regularities within the vast prior literature on emotional memory, the CMEM provides a theoretical context within which to situate these effects by contextualizing them within a framework that provides explanations for key processes such as the attribution of valence and arousal to external stimuli and the experience of discrete emotion as resulting from emotion construction. At the same time, the critical value of the CMEM is seen in its ability to make several novel predictions regarding the effects of emotion on memory (see Table 1). In particular, the CMEM predicts that (a) the availability, accessibility, and use of emotion concepts during emotion construction will bias the way that stimuli are remembered; (b) the effects of valence, arousal, and discrete emotions will depend on the availability of conceptual emotion knowledge; and (c) variability in emotion construction at the situational, individual, and cultural levels will influence how emotions affect memory.

We believe that by incorporating mechanistic theories from affective science that speak to the nature of emotion, such as the theory of constructed emotion, memory researchers will be able to generate new and exciting hypotheses regarding the influence of emotion on memory. In addition to informing basic cognitive science in this way, the CMEM has the potential to inform applied work in areas including eyewitness testimony (where features of the situation, individual, or culture might influence how these events, which are often highly emotional, are later remembered in the courtroom) and disorders characterized by intrusive emotional memories (e.g., post-traumatic stress disorder). It is our hope that the CMEM will prove fruitful in guiding future research, either by motivating researchers to test its predictions, or by motivating researchers to specify and test competing models.

## Transparency

*Action Editor:* Moira R. Dillon

*Editor:* Arturo E. Hernandez

## Notes

1. As pointed out elsewhere (Barrett, 2015), the circumplex model of affect was created to describe the affective dimensions underlying emotion rather than the processes by which emotions are generated and may therefore be thought of as a descriptive model rather than a mechanistic theory of emotion.

2. Although emotion tends to result in trade-offs for peripheral details within a single episode, the impact of emotion on other aspects of associative memory, such as memory for temporal order, is currently subject to lively debate (for a review, see Petrucci & Palombo, 2021). Although some have found that emotion reduces temporal associative memory (Hurlemann et al., 2005), others have found that emotion either does not impact (de Montpellier & Talmi, 2025) or enhances temporal memory (Clewett & McClay, 2024; Dev et al., 2022; Riegel et al., 2025). Given that the role of emotion on associative memory, generally speaking, is still unclear, we have restricted our conclusions about the general impacts of emotion on memory to the relatively well-established effects on memory for peripheral contextual details within isolated episodes.

3. Although not all appraisal theories assume that specific appraisal patterns are consistently associated with specific discrete emotions and response patterns (e.g., Clore & Ortony, 2013), such appraisal theories have not yet been applied to episodic memory research.

4. Elsewhere, categorization has been referred to as *conceptualization* (e.g., Lindquist, 2013) or *situated conceptualization* (Wilson-Mendenhall et al., 2011).

5. Although some have claimed to find evidence of emotion-specific patterns of physiology by using pattern-classification approaches (e.g., Kragel & LaBar, 2013, 2016; Stephens et al., 2010), others have argued that such patterns represent abstract statistical summaries rather than the context-free physiological states necessary to classify a given instance as an exemplar of a given emotion across contexts (Barrett, 2017a; Bonar & Lindquist, 2025; Clark-Polner et al., 2016; Siegel et al., 2018). This conceptualization of emotion categories as populations of variable category instances begs a major question in affective science of how much structure exists in the world (i.e., in the raw sensory information from the external environment or one’s body) versus how much is imposed via conceptual knowledge that is shared among members of a culture. It is beyond the scope of this article to answer this question in the depth it deserves, but existing evidence seems to suggest that within some domains (e.g., autonomic responding during emotion; Hoemann et al., 2020; McVeigh et al., 2024), structuring of the underlying data by emotion category is quite small and is instead a product of emotion category, situational factors, and idiographic factors. Even within the domain of self-report, there is great variability in the emotion category participants assign to an emotional stimulus (Westlin et al., 2026). A goal of future constructionism-inspired work is to measure how much structure exists in people’s ecologically valid emotional experiences and model sources that can influence any structure that does exist.

6. Although emotion concepts might be thought of as similar to schemas in that both are representations of conceptual knowledge, constructionist theories tend to conceive of emotion concepts as flexible, contextually sensitive constructions composed of prior category exemplars rather than as rigid, abstract knowledge structures that are stored in long-term memory (Lindquist & Barrett, 2008b; Wilson-Mendenhall et al., 2011).

7. The theory of constructed emotion proposes that the same processes that underlie the perception of emotion in others also give rise to the experience of emotions within oneself (i.e., the categorization of sensory information using situated emotion concepts; Barrett, 2014). Consequently, disruptions in emotion perception can be seen as disruptions in emotion construction.

8. Similar disruptions to emotion perception have also been demonstrated in patients with *semantic aphasia* (Souter et al., 2021), a condition characterized by deficits in the retrieval of conceptual knowledge (see also Foley et al., 2021, for a case report demonstrating disrupted emotion perception in an individual with tumor-related anterior temporal lobe damage).

9. Although semantic satiation has been shown to disrupt the availability of conceptual knowledge about discrete emotions generally (Gendron et al., 2012; Lindquist et al., 2006), it does not appear to do so in an emotion-specific manner (Lindquist et al., 2006, Study 3). Thus, the semantic satiation of a given emotion word such as “anger” is predicted to result in the general inhibition of discrete emotional-memory effects rather than inhibiting the mnemonic effects of anger specifically.

10. Emotional granularity is sometimes referred to as “emotion differentiation” (Hoemann, Nielson, et al., 2021).

11. As pointed out by a reviewer, investigating the determinants of false memory for emotional Deese-Roediger-McDermott (DRM) word lists created using semantic associates of discrete emotion concepts (e.g., fear) may shed light on the impact of conceptual emotion knowledge on emotional memory. For instance, it might be predicted that whereas highly granular individuals will show increased false memory for discrete emotion words such as “fear,” as a result of having stronger conceptual representations of such discrete emotion concepts and their associates, individuals low in granularity or high in traits such as alexithymia may show reduced false memory for discrete emotions. Because only two of Chang et al.’s (2021) 32 emotional DRM lists have discrete emotion concepts as lures (anger and love), the first author’s dissertation examining the relationship between granularity and emotional memory focused on false memory for emotional things (e.g., needles) and descriptors (e.g.., “dead”). With these considerations in mind, research within this area may benefit from the development of DRM lists based on discrete emotion concepts.

12. In addition to West (2023), we are aware of only a small number of prior investigations in which memory was predicted as a function of participants’ idiosyncratic affective ratings rather than as a function of normative ratings (e.g., Faul et al., 2025; Mickley Steinmetz et al., 2018). However, because these investigations did not directly compare the predictive validity of normative and idiosyncratic ratings using comparable statistical models, as in West (2023), these investigations do not speak to whether idiosyncratic ratings predict memory with greater validity than normative ratings, as predicted by the CMEM and suggested by West (2023).

13. Even so, because cultures without words for a superordinate emotion state may still have words for subordinate affective states, like “good” and “bad,” it may be that some degree of interrelatedness might still be observed for normatively emotional stimuli in the absence of an overt concept for emotional states. Researchers in this area are encouraged to consider the existence of such subordinate conceptual categories when generating predictions regarding the organization of emotional stimuli within a specific cultural context.
